# An Optical Probe for Real-Time Monitoring of Self-Replicator Emergence and Distinguishing between Replicators

**DOI:** 10.1021/jacs.1c11594

**Published:** 2022-02-09

**Authors:** Joydev Hatai, Yigit Altay, Ankush Sood, Armin Kiani, Marcel J. Eleveld, Leila Motiei, David Margulies, Sijbren Otto

**Affiliations:** † Centre for Systems Chemistry, Stratingh Institute, University of Groningen, Nijenborgh 4, 9747 AG Groningen, The Netherlands; ‡ Department of Chemical and Structural Biology, Weizmann Institute of Science, Rehovot 7610001, Israel

## Abstract

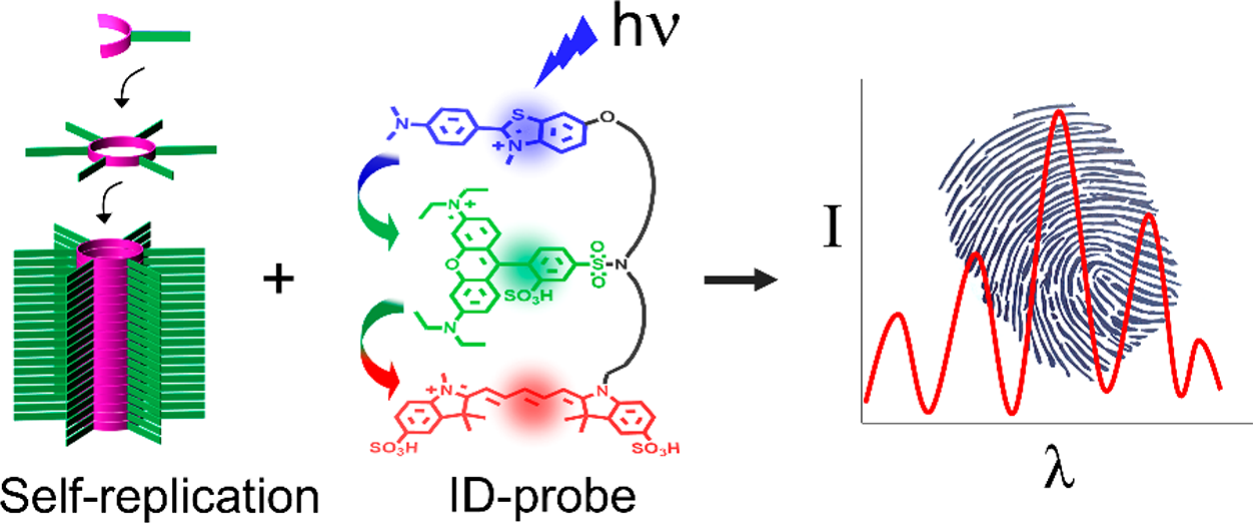

Self-replicating systems play an important role in research on the synthesis and origin of life. Monitoring of these systems has mostly relied on techniques such as NMR or chromatography, which are limited in throughput and demanding when monitoring replication in real time. To circumvent these problems, we now developed a pattern-generating fluorescent molecular probe (an ID-probe) capable of discriminating replicators of different chemical composition and monitoring the process of replicator formation in real time, giving distinct signatures for starting materials, intermediates, and final products. Optical monitoring of replicators dramatically reduces the analysis time and sample quantities compared to most currently used methods and opens the door for future high-throughput experimentation in protocell environments.

## Introduction

**Figure 1:**
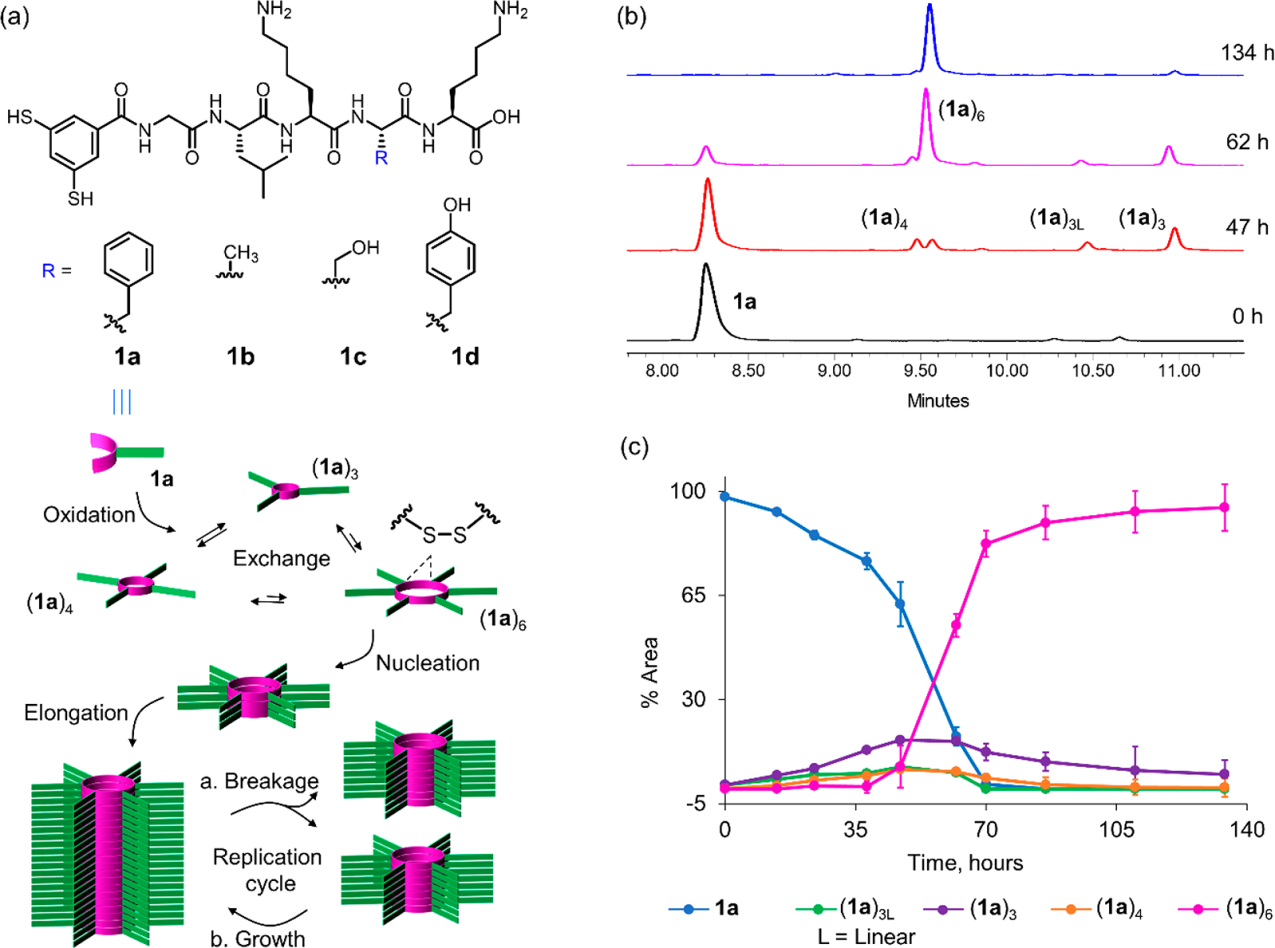
(a) Mechanism of self-assembly-driven self-replication. Air oxidation of dithiol building block **1a** initially produces a mixture of disulfide macrocycles of different ring sizes that interconvert through thiol–disulfide exchange. Assembly of, in this case, the cyclic hexamer (**1a**)_6_ into fibers results in the autocatalytic production of more hexamer. Fiber formation is driven by a combination of π-stacking interactions and the assembly of the peptide tails into β-sheets. Agitation-induced fiber breakage liberates more growing fiber ends, enabling exponential growth.^37^ (b) Selected ultraperformance liquid chromatography (UPLC) traces (monitored at 254 nm) recorded at different stages during the emergence of replicator (**1a**)_6_. (c) Kinetic profile (average of three independent experiments) of the emergence of (**1a**)_6_. Samples were made from 30 μM building block **1** in 50 mM (in boron atoms) borate buffer, pH 8.2, stirred at 1200 rpm at 30 °C.

The question how life can emerge from an abiotic chemical mixture is among the grand challenges in contemporary science.^1−4^ Self-replicating systems play a key role in addressing this question.^5−7^ In the last decades, different self-replicating systems have been reported based on synthetic molecules^8−10^ or biology-inspired motifs such as nucleobases^11^ or peptides^12−16^ or combinations thereof.^17−19^ Self-replication can be achieved through a template-based mechanism, modeled after the replication of nucleic acids in nature, or driven by self-assembly, as shown schematically in Figure 1a.^20^

The field of self-replication is now gradually entering the next phase in which systems of replicators are extended to capture additional essential ingredients for life, including proto-metabolism and compartmentalization.^7,21,22^ Also efforts directed at achieving Darwinian evolution of these systems are imminent. This shift in focus in research on self-replicating systems will put new demands on analytical tools.

Until now the study of self-replicators has relied on analytical methods such as NMR or chromatography (HPLC/UPLC) coupled to mass spectrometry. While these techniques have proven very powerful in unravelling the behavior of systems of individual replicators in solution, they are less suitable for parallel screening, sampling of small volumes and low concentrations (in the case of NMR), and in situ monitoring (in the case of chromatography). Thus, for further development of self-replicators in the direction of life, additional analytic tools are required that would ideally allow real-time and nondestructive monitoring of self-replicators at low concentrations and in small sample volumes.

Fluorescent molecular probes appear particularly suitable for monitoring self-replication and have been used in a few instances. Philp et al. have labeled a replicator precursor with a fluorophore to monitor a propagating replication–diffusion front.^23^ Ashkenasy et al. covalently labeled a peptide-based replication system with a fluorophore, enabling monitoring self-replication through the extent of fluorescence quenching.^24^ We have used thioflavin T (ThT) to monitor β-sheet formation in our peptide-based replicators.^25^ However, none of the probes used so far allow for discrimination between different replicators.

To track the dynamic formation of several molecular species in solution, a new class of fluorescent molecular probes,^26−30^ termed ID-probes,^29^ was recently developed. Unlike conventional small-molecule-based probes, which generally bind a single analyte and produce a single fluorescence output, ID-probes combine several fluorophores and nonspecific (or partially specific) recognition elements that enable them to interact with different molecular species in a mixture and generate unique identification (ID) fingerprints for different analytes and their combinations. This differential sensing mode is similar to that underlying the function of the olfactory system or artificial nose/tongue analogues.^31−36^

It occurred to us that the ID-probe strategy could provide an important new tool to study self-replicating systems, one that would complement the current analytical techniques.

**Figure 2:**
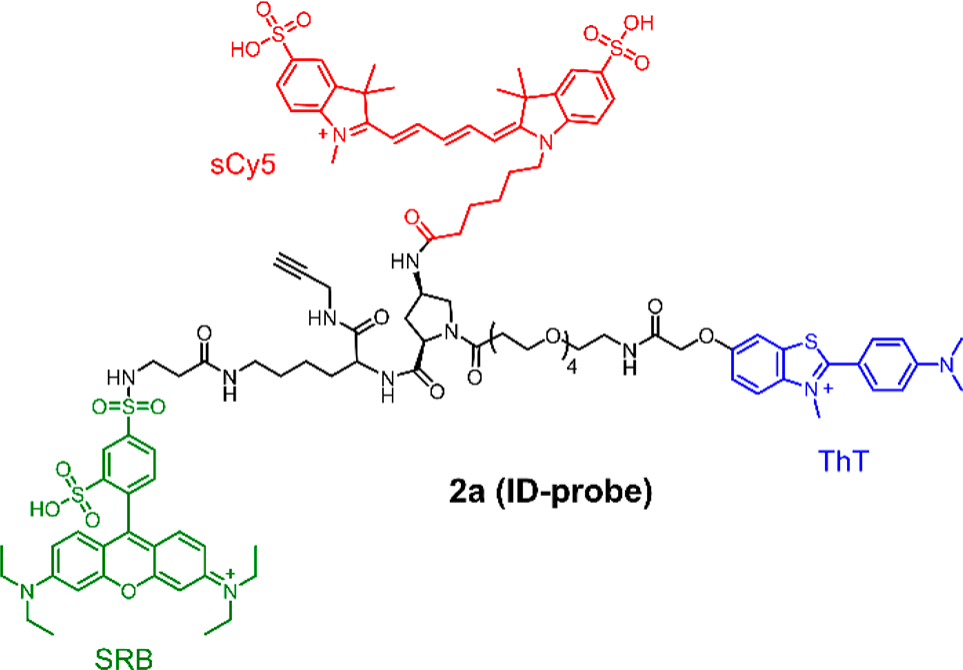
Structure of the pattern-generating ID-probe **2a** carrying three fluorescent reporters: ThT (blue, Ex: 440 nm, Em: 490 nm), SRB (green, Ex: 530 nm, Em: 595 nm), and sCy5 (red, Ex: 630 nm, Em: 675 nm).

We now report the design and synthesis of an ID-probe developed to study the self-replicating systems made from a family of dithiol-containing peptides (**1a**–**d**, Figure 1a; typical analysis by UPLC shown in Figure 1b and c). We first show that a specific pattern-generating probe (**2a**, Figure 2) can be used to discriminate between building blocks, intermediates, and final replicators prepared separately. We subsequently used the sensor to monitor the process of replicator formation in situ in real time. Finally, we show that the sensor can discriminate between replicators of subtly different chemical nature.

## Results and Discussion

### Probe Design

We designed ID-probe **2a** to monitor systems of self-replicators made from the family of peptide-containing building blocks (such as **1a**–**d**) that we developed recently.^38,39^ Replication is partially driven by assembly of the peptides into β-sheets (see Figure 1a). We exploit this feature by including ThT in the design of the sensor, which is well known to interact with β-sheets, resulting in a marked increase in its fluorescence that depends only little on the exact nature of the β-sheet assembly.^40,41^ In order to enhance the discriminating ability of the sensor, we also introduced two additional dyes: sulforhodamine B (SRB) and sulfo-Cy5 (sCy5). These dyes were selected based on their successful use in a previously developed ID-probe that was used to study amyloid beta (Aβ) aggregates (like our replicators, these aggregates involve extensive β-sheet formation).^28^ The dyes can engage in Förster resonance energy transfer (FRET) with each other (see Figure S1 for the absorption and emission spectra of the individual dyes). They are also likely to bind to the replicator systems through electrostatic and hydrophobic interactions. We expect that the exact geometry of binding should be different for different peptide assemblies, causing differences in the optical properties of the individual dyes as well as in the efficiency of FRET, which is highly dependent on the distance between the fluorophores. The three dyes were attached on a *cis*-aminoproline core that projects them in the same direction, which should benefit FRET. We chose a short ethylene oxide spacer for one of the probes to enhance the water solubility. The flexible spacers between core and dyes allow the sensor to adopt different binding geometries when bound to different analytes, which should result in different optical signatures. We speculate that this will lead to a collection of bound states in which the dyes experience different microenvironments and have different distances to each other. The optical response will be a sum of all these states and is likely to differ for different analytes. Finally, the sensor was equipped with an alkyne group to enable easy future structural elaboration using click chemistry.

### The ID-Probe Response Involves FRET

**Figure 3:**
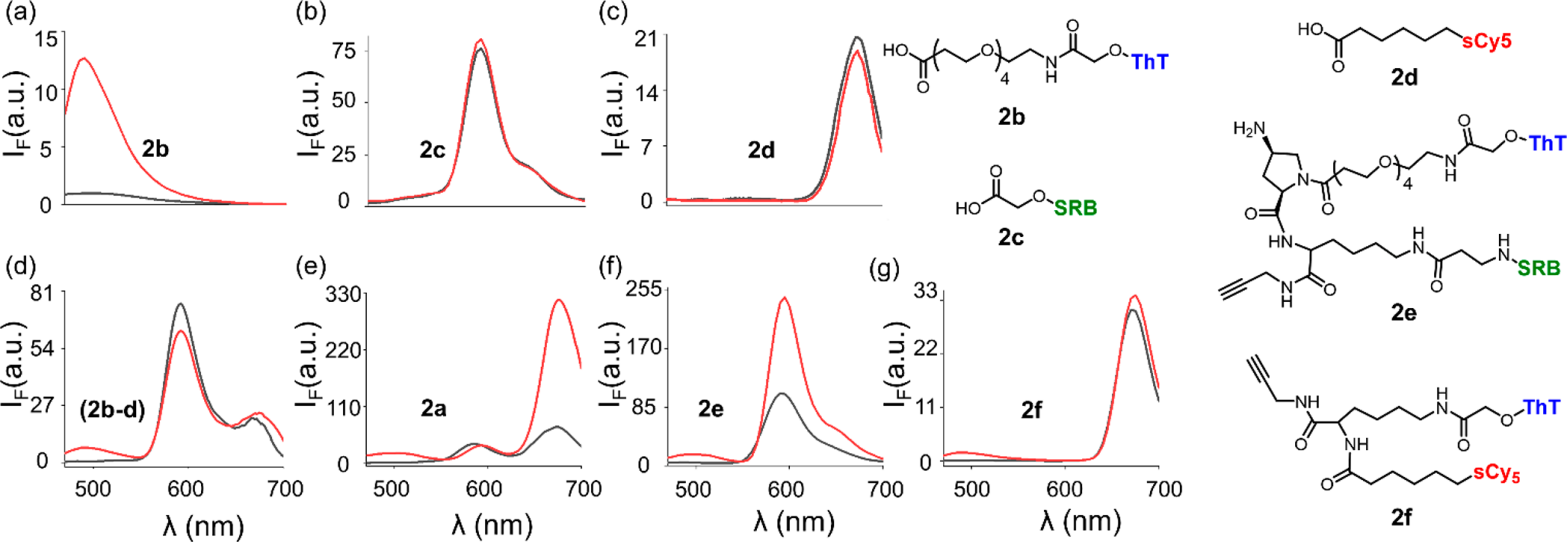
Emission spectra (λ_ex_ = 440 nm) of control molecules (a) **2b**; (b) **2c**; (c) **2d**; and (d) an equimolar mixture of **2b**, **2c**, and **2d**. Emission spectrum of (e) probe **2a** and that of different binary dye conjugates (f) **2e** and (g) **2f**. Conditions: 2.0 μM dye construct(s), 50 mM borate buffer (pH 8.2), in the absence (black line) and presence (red line) of fibers of replicator (**1a**)_6_ (30 μM in building block **1a**). The fluorescence intensity shown on the *y* axes is normalized against the emission intensity of **2b** at 490 nm in buffer.

A key hypothesis underlying the design of **2a** is that the combination of the three dyes on a single molecular platform would result in FRET between the dyes. This optical communication should enable sCy5 and SRB to respond to the presence of replicators, despite the fact that the emission of these two dyes, when used in isolation, is unlikely to be affected by the presence of the self-replicators. To test this hypothesis, we first measured the fluorescence emission generated by a derivative of each dye (Figure 3a–c) and by a mixture containing the three derivatives (Figure 3d) in the absence (black line) and presence (red line) of replicator (**1a**)_6_. The resulting spectra were compared to one generated by **2a** under the same conditions (Figure 3e). Inspecting the fluorescence responses of the individual dyes (Figure 3a–c) revealed that, as expected, only ThT responded with a marked (13-fold) increase in fluorescence (Figure 3a, Em: 490 nm) upon irradiation at 440 nm, whereas the emission of SRB (Figure 3b, Em: 595 nm) or sCy5 (Figure 3c, Em: 675 nm) remained unchanged. Moreover, when the individual dyes were combined in a single solution (Figure 3d), fluorescence enhancement was almost exclusively observed in the ThT channel, precluding the possibility of FRET between the dyes in the absence of a covalent linkage between them. In contrast to the strong, single-channel response of the dye mixture (Figure 3d, ThT channel), in the presence of the (**1a**)_6_ fibers, **2a** exhibited notable changes in both the ThT and sCy5 channels (Figure 3e), indicating FRET between the three dyes.

The prominent increase in the emission of sCy5 (Ex: 630 nm, Em: 675 nm) upon excitation of ThT (Ex: 440 nm, Em: 490 nm) suggests that FRET is mediated via the SRB dye, which serves as a FRET acceptor for ThT and as a donor for sCy5. This assumption was validated by comparing the fluorescence response of compound **2e**, which combines ThT and SRB (Figure 3f), to the response of **2f** that bears ThT and sCy5 (Figure 3g). In agreement with an SRB-mediated FRET process, compound **2e** exhibited a large increase in the emissions of both ThT and SRB in the presence of (**1a**)_6_, whereas in compound **2f**, mainly the emission of ThT was enhanced. This indicates that the SRB is essential for obtaining efficient optical communication between the three dyes and that their integration on a unimolecular platform is required for the generation of fluorescence patterns.

### The Sensor Can Discriminate between Different Replicators and Their Precursors

To test the discriminatory ability of **2a**, we first subjected it to separately prepared samples that represent different phases during the emergence of the self-replicators: the starting monomers **1a**, a mixture dominated by trimers and tetramers (**1a**)_3_/(**1a**)_4_, which are intermediates in replicator formation that are rapidly interconverting (see Figure 1a), and replicator fibers (**1a**)_6_ (prepared as described in the Methods section). The individual samples were characterized by liquid chromatography–mass spectrometry (UPLC-MS; Figure S2). Analysis of replicator (**1a**)_6_ by transmission electron microscopy (TEM) and ThT assay confirmed the expected presence of fibers and β-sheets, respectively (Figure S3).

Fluorescence was measured using a microplate reader in 384-well microplates. The fluorescence spectra were recorded by mixing **2a** (2.0 μM) with different samples (e.g., monomers, mixtures of trimers–tetramers, and fibers; 30 μM in units of building block) prepared from building block **1a** in borate buffer (50 mM in boron atoms, pH 8.2).

**Figure 4:**
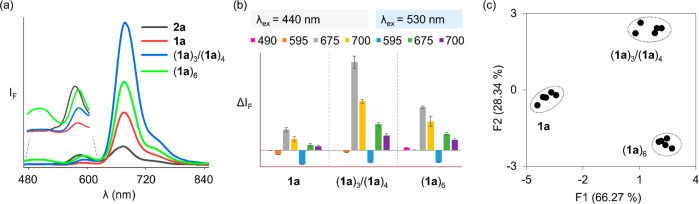
(a) Emission spectra of **2a** (2.0 μM, 50 mM borate buffer, pH 8.2) in the presence of monomer **1a**, a mixture of trimers and tetramers (**1a**)_3_/(**1a**)_4_, or replicator fibers (**1a**)_6_, all at a concentration of 30 μM in units of **1a**. (b) Change in fluorescence intensity of **2a** at different emission channels (λ_ex_ = 440 and 530 nm, respectively) upon being exposed to **1a**, (**1a**)_3_/(**1a**)_4_, or replicator fibers (**1a**)_6_. (c) Principle component analysis (PCA) of the fluorescence data in (b) showing five repeats for each sample.

Inspecting the fluorescence responses of **2a** to the different samples upon excitation of the ThT dye (λ_ex_ = 440 nm) revealed a markedly different pattern for each sample (Figure 4a). In the presence of intermediates (**1a**)_3_/(**1a**)_4_, we observed a decrease in the emission of the SRB (595 nm) channel and an increase in the emission of sCy5 (675 nm). Unexpectedly, sCy5 fluorescence was also enhanced in the presence of monomers **1a**. Fluorescence of sCy5 most likely comes about through FRET, which seemed unlikely to be promoted by binding to molecularly dissolved **1a** (given the relatively small size of this molecule) and suggests that this building block (and potentially also the (**1a**)_3_/(**1a**)_4_ mixture) forms aggregates. Indeed, titration of **2a** (2.0 μM) with **1a** or (**1a**)_3_/(**1a**)_4_ revealed the typical signature of a critical aggregation concentration (CAC) where CAC_**1a**_ = 9.2 μM (Figure S4a). Previous studies on the (**1a**)_3_/(**1a**)_4_ mixture had already revealed that these molecules also aggregate.^42^ Studies with our ID-probe confirm this conclusion (CAC_(**1a**)3/(**1a**)4_ = 5.1 μM in units of **1a**, Figure S4b).

In contrast to the (**1a**)_3_/(**1a**)_4_ intermediates, which induced a decrease in the SRB channel and an increase in the sCy5 channel, in the presence of (**1a**)_6_ the emission of **2a** was increased in both the ThT (440 nm) and the sCy5 (675 nm) channels, resulting in a unique fluorescence fingerprint for the replicator fibers.

To enhance the differentiation ability, we measured the optical response of **2a** to the different oligomers of **1a** at different emission channels: 490, 540, 570, 595, 640, 675, and 700 nm upon excitation of ThT (440 nm) and 570, 595, 640, 675, and 700 nm upon excitation of SRB (530 nm). Figure 4b shows the resulting data for a selection of these channels, while all data were used for a principal component analysis (PCA; Figure 4c). PCA is a linear transformation data processing technique that is commonly used to reduce the dimensionality of multidimensional data sets enabling its visualization. It chooses a linear combination of data (the principal components; here a selection of fluorescence intensities at specific wavelengths) that maximizes the spread of the data in a two-dimensional graph.^43^ The PCA map clearly shows that **2a** can discriminate between the monomers, trimer/tetramers, and fibers made from **1a**. Using this PCA map an untrained student^28,29^ was able to identify with 100% accuracy 12 samples of which the student did not know the composition (Figure S5 and Table S3).

In order to probe the sensitivity of the technique, we repeated the PCA at different analyte concentrations (10 μM, 3.0 μM, and 300 nM in units of **1a**). The corresponding data are shown in Figure S6 and indicate that the sensor’s discriminatory ability gradually diminishes as analyte concentrations are lowered, but is still present even at high nanomolar concentrations.

These experiments were run in 384-well microplates using a sample volume of 45 μL per well, substantially increasing sample throughput and reducing sample volumes, compared to previously used methods for monitoring self-replicators.

### The Sensor Enables Monitoring Self-Replicator Formation in Situ

As a first test of the suitability of the ID-probe to track the emergence and growth of replicators in real time, where the molar ratio of precursors (**1a**)_3_/(**1a**)_4_ and replicators (**1a**)_6_ dynamically changes, the emission of **2a** was recorded upon exposure to mixtures containing different molar ratios (specified in Table 1) of separately prepared (**1a**)_6_ and (**1a**)_3_/(**1a**)_4_. The compositions of these mixtures represent different stages of replicator emergence (Figure 1b,c). PCA of the resulting data shows that **2a** can discriminate between different molar ratios of precursors and replicators (Figure S7).

**Table 1:** Composition of Samples Prepared by Mixing Different Molar Ratios of Fibers (**1a**)_6_ to Trimers–Tetramers (**1a**)_3_/(**1a**)_4_

	sample composition (%)^a^	
sample	trimers–tetramers (**1a**)**_3_**/(**1a**)_**4**_	fibers (**1a**)_**6**_
1	100	0
2	80	20
3	60	40
4	30	70
5	0	100

a The total concentration of each sample was 30 μM in units of building block **1a**. These samples represent different stages of replicator emergence.

**Figure 5:**
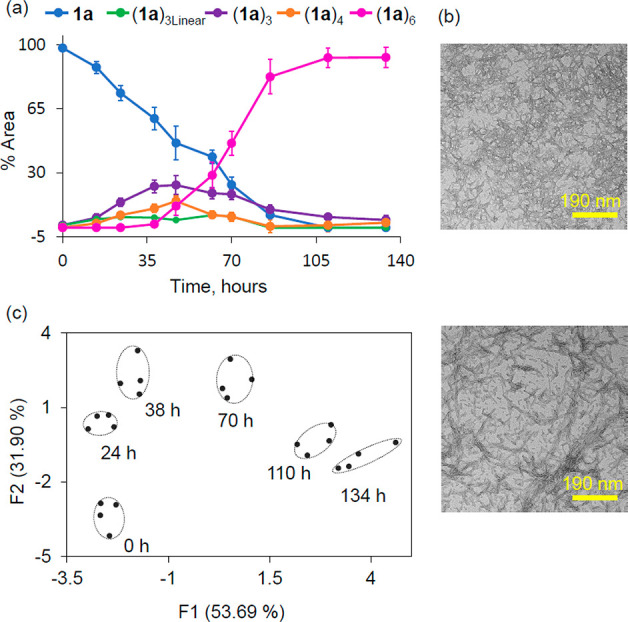
(a) UPLC analysis of the change in product distribution (average of three independent experiments) in a mixture made from building block **1a** (30 μM in building block **1a**) co-incubated with sensor **2a** (2.0 μM) in borate buffer (50 mM, pH 8.2, stirred at 1200 rpm at 30 °C). (b) TEM images of fibers of replicator (**1a**)_6_ in the absence (top) and presence (bottom) of sensor **2a**. (c) PCA of the emission data recorded for the same sample at various time points.

Encouraged by these results, we investigated whether ID-probe **2a** could be used to track the spontaneous emergence of replicators from **1a** in situ and in real time (Figure 5). To this end, we co-incubated building block **1a** (30 μM) and sensor **2a** (2.0 μM) and followed the changes in the emission of the ID-probe over time (Figure S8). To confirm that the presence of the ID-probe does not affect the dynamic formation of the different replicators in the mixtures, we analyzed the replication process and its kinetics by previously established techniques (UPLC-MS, TEM). The UPLC-based kinetic profile obtained in the presence of **2a** (Figure 5a) is comparable to one acquired without the ID-probe (Figure 1c). The small differences between the data in these two figures is similar in magnitude to the differences we typically observe in the emergence of replicators in experiments conducted at different times. TEM images (Figure 5b) revealed that similar (**1a**)_6_ fibers were obtained in the absence (top) and presence (bottom) of the probe.

Analyzing the patterns generated by **2a** over time (Figure S8) by PCA (Figure 5c) shows that the ID-probe allows for qualitative tracking of the growth of replicators in real time in situ (data for additional time points are shown in Figure S9). A control experiment in the absence of replicators revealed that the emission of sensor **2a** remained unchanged over time (Figure S10), confirming that the observed changes in fluorescence patterns resulted from changes in the composition of the mixture. In principle one could envisage that the probe may also find use in quantitative kinetic studies, but this will require enhancing the accuracy with which it reports on small differences in composition.

### The ID-Probe Can Discriminate between Different Self-Replicators

We tested whether ID-probe **2a** could also differentiate between replicators that are generated from distinct peptide monomers: **1b**, **1c**, and **1d** (Figure 1a). The only structural difference between these monomers and **1a** is that phenyl alanine in **1a** was replaced with another amino acid: alanine (**1b**), serine (**1c**), or tyrosine (**1d**). Another difference concerns the self-assembled structures that are formed during the replication process. Unlike **1a**, which yields hexamers, **1b** and **1c** form octamers (**1b**)_8_ and (**1c**)_8_, respectively, whereas **1d** generates pentamers (**1d**)_5._^38,39^ The formation of these pentamers was unexpected, given that in previous work (conducted at a higher building block concentration of 3.8 mM and RT instead of 2.0 mM and 45 °C) different sized replicators were formed. However, as we noted in our previous work, **1d**-based replicators show an unusual plasticity in ring size._._^39^

**Figure 6:**
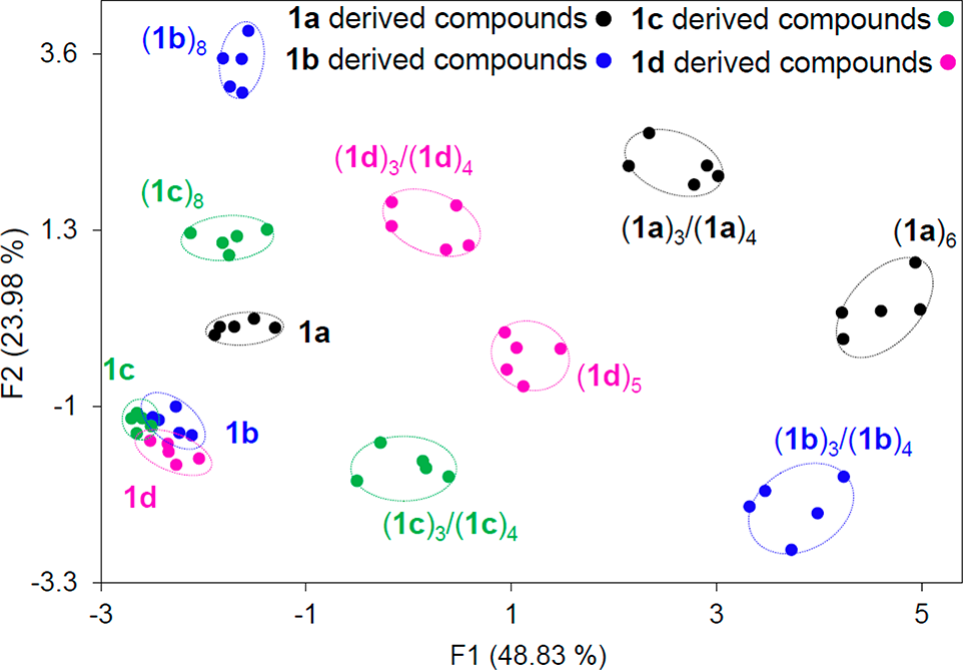
PCA of the emission patterns generated by **2a** (2.0 μM) at different emission channels (λ_ex_ = 440 and 530 nm, respectively) in the presence of monomers, a mixture of trimers–tetramers, and replicator fibers prepared from building blocks **1a**–**d** (30 μM in building block). The fluorescence data for each sample consist of five repeats.

Fibers of self-replicators derived from **1a**–**d** (2.0 mM) were prepared by agitating solutions that were subjected to slow air oxidation at 30 °C for (**1a**)_6_ or fast partial oxidation (50%) by sodium perborate followed by air oxidation at 45 °C^38,39^ (see Figures S2, S11–S13 for UPLC-MS characterization). Analysis by ThT emission and TEM (Figures S3, S14–S16) confirmed the formation of β-sheets and fibers, respectively (as observed previously for these systems^38,39^). Samples dominated by trimers and tetramers (as mixture) of these building blocks were also prepared by rapidly oxidizing the individual monomers (2.0 mM) with sodium perborate (40 mM, Figures S2, S11–S13 for UPLC-MS characterization). The different replicators and their small-ring precursors made from building blocks **1a**–**d** (30 μM each) were then incubated with **2a** (2.0 μM), and their fluorescence emission spectra were recorded (Figure S17). The PCA of these data (Figure 6) clearly shows that **2a** can discriminate between the differently sized macrocycles (trimers and tetramers) and replicators made from the different building blocks. Yet, the probe has difficulty in discriminating between the monomers from which these systems are built up. Using the PCA map shown in Figure 6 an untrained student was able to correctly identify 26 samples out of 28 of which the composition was unknown to the student (Figure S18, Table S4), demonstrating the utility of the ID-probe for the rapid analysis of samples of replicators in a nondestructive way.

## Conclusions

We have demonstrated the use of a pattern-generating fluorescent molecular probe for straightforwardly detecting, discriminating between, and real-time tracking of peptide-based self-replicators. In systems dominated by a single replicator the sensor is able to discriminate between replicators with different macrocycle sizes (e.g., hexamers, octamers, pentamers) and amino-acid composition. It is also able to differentiate macrocycles with the same size but subtly different peptide sequences. Furthermore, the conversion of building block into replicator could be monitored qualitatively in situ and in real time without interfering with the replication process. The probe was also found to respond differently to different aggregates formed by the building block and the replicator precursors, indicating that its discriminating ability extends beyond the fibrous aggregates for which it was designed. This new technology for the optical analysis of self-replicating systems opens the door for continuous monitoring of parallel experiments in high-throughput ways in small volumes (i.e., in microdroplets or other protocell environments), enabling, for example, the study of stochastic effects, which may prove important in the emergence and evolution of new forms of life. Efforts in this direction are currently underway in our laboratory, which will also reveal the effects of protocell boundary and environment on the probe’s response. The work builds one of the first bridges between the still poorly connected fields of systems chemistry and differential sensing, demonstrating the power of such optical tools for rapid fingerprinting of complex reaction networks in terms of composition and dynamics.

## Methods

### Preparation of Monomer Solutions

Building block **1a**, **1b**, **1c**, or **1d** (stored at −20 °C) was added (around 2 mg) to an HPLC vial (12 × 32 mm), and around 1.0 mL of freshly prepared 50 mM (in boron atom) borate buffer (pH 8.2) was added. The pH of the solution was measured and, if necessary, adjusted using 1 M NaOH solution. The final concentration of stock solution was 2.0 mM, and the monomer solutions were used immediately for the fluorescence experiments. For UPLC and LC-MS analysis, 10 and 5 μL of sample was injected, respectively, from the solution prepared by mixing 10 μL of stock solution (2.0 mM) and 80 μL of UPLC grade water (Figures S2, S11–S13).

### Preparation of Mixtures of Trimers–Tetramers (**1a**)_**3**_**/**(**1a**)_**4**_, (**1b**)_**3**_**/** (**1b**)_**4**_, (**1c**)_**3**_**/**(**1c**)_**4**_, and (**1d**)_**3**_**/**(**1d**)_**4**_

Building block **1a**, **1b**, **1c**, or **1d** (stored at −20 °C) was added (around 2 mg) to an HPLC vial (12 × 32 mm), and around 1.0 mL of freshly prepared 50 mM (in boron atoms) borate buffer (pH 8.2) was added. The pH of the solution was measured and, if necessary, adjusted using 1 M NaOH solution. The final concentration of stock solution was 2.0 mM in borate buffer (50 mM, pH 8.2). A sodium perborate solution (40 mM) was prepared freshly in borate buffer. To prepare a mixture dominated by trimers and tetramers, individual monomer solutions (500 μL, 2.0 mM) were fully oxidized using sodium perborate (25 μL, 40 mM) solution. The sample was thoroughly mixed using a micropipet and stored at room temperature for 1 h. Purity and composition of the samples were determined by UPLC and UPLC-MS, respectively. The resulting stock solutions can be stored at 25 °C for 2 to 3 days without observing any notable changes in sample composition. For UPLC and LC-MS analysis, 10 and 5 μL of sample was injected, respectively, from the solution prepared by mixing 10 μL of stock solution (2.0 mM) and 80 μL of UPLC grade water (Figures S2, S11–S13).

### Preparation of Replicator Fibers (**1a**)_6_, (**1b**)_8_, (**1c**)_8_, and (**1d**)_5_

Building block **1a**, **1b**, **1c**, or **1d** (stored at −20 °C) was added (around 2 mg) to an HPLC vial (12 × 32 mm) containing a Teflon-coated magnetic stirring bar (5 × 2 mm, VWR), and around 1.0 mL of freshly prepared 50 mM [in boron atoms] borate buffer (pH 8.2) was added. The pH of the solution was measured and, if necessary, adjusted using 1 M NaOH solution. The final concentration of stock solution was adjusted to 2.0 mM. A sodium perborate solution (40 mM) was prepared in freshly prepared borate buffer (50 mM, pH 8.2) solution. To prepare fibers, individual monomer solutions (500 μL, 2.0 mM) were partially oxidized (50%) using sodium perborate (12.5 μL, 40 mM) solution. The sample was thoroughly mixed using a micropipet, and the vial was subsequently closed with a Teflon septum screw cap. The mixture was then placed in a metallic HPLC vial holder and stirred at 1200 rpm at 45 °C for 7 days. The composition of the mixture was periodically analyzed by UPLC and UPLC-MS. For UPLC and LC-MS analysis, 10 and 5 μL of sample was injected, respectively, from the solution prepared by mixing 10 μL of stock solution (2.0 mM) and 80 μL of UPLC grade water (Figures S2, S11–S13). To confirm the formation of fibers, emission spectra of ThT (2 μM, 50 mM borate buffer in boron atoms) in the presence of different samples (30 μM building block **1**) prepared from peptide building block **1a**, **1b**, **1c**, or **1d** were recorded (Figures S3, S14–S16).

### Fluorescence Measurements Differentiating among the Monomers **1a**–**d**, Mixtures of Trimers–Tetramers (**1a**)_3_/(**1a**)_4_, (**1b**)_3_/(**1b**)_4_, (**1c**)_3_/(**1c**)_4_, and (**1d**)_3_/(**1d**)_4_, and Replicator Fibers (**1a**)_6_, (**1b**)_8_, (**1c**)_8_, and (**1d**)_5_

Fluorescence was measured using a BioTek Synergy H1 microplate reader, in black flat-bottom polystyrene 384-well microplates (Greiner). A 200 μM stock solution of **2a** was prepared in anhydrous DMSO. The fluorescence spectra were recorded within 1 h of incubation of **2a** (2.0 μM) individually with different samples (30 μM in units of building block, e.g., monomers, mixtures of trimers–tetramers, and fibers) prepared from building block **1a**, **1b**, **1c**, or **1d** in borate buffer (50 mM in boron atoms, pH 8.2) at 25 °C. These experiments were performed in five replicates where each sample was freshly prepared starting from the weighing step. The spectra before addition of samples were subtracted from those recorded after the addition of different samples. Principle component analysis (XLSTAT 2020.5.1) was applied to discriminate the emission patterns obtained at the following excitation and emission wavelengths: λ_ex_: 440 nm, λ_em_: 490, 540, 570, 595, 640, 675, 700 nm; and λ_ex_: 530 nm, λ_em_: 570, 595, 640, 675, 700 nm (Figure 4c and Figure 6). In the PCA analysis the emission data and sample names are considered as observations/variables and observation labels, respectively. The PCA analysis is performed on the correlation matrix, to ensure that large differences in magnitude of emission channel responses do not skew the analysis. In the outputs and charts tab, the descriptive statistics and correlation charts are selected and the filtering option is unchecked. The PCA plot was constructed using the percentage of variability represented by the first two factors (F1 and F2 axes).

The discrimination efficiency of the system was further validated by identifying (Figure S5, Table S3 and Figure S18, Table S4) unknown samples that were freshly prepared from building block **1a**, **1b**, **1c**, or **1d** on different days. Unknown samples were analyzed using the XLSTAT (version 2020.5.1) prediction mode according to the posterior probabilities for each blind test.

### Preparation of Libraries of Mixtures Containing Different Molar Ratios of (**1a**)_**6**_ and (**1a**)_**3**_/(**1a**)_**4**_

Analyte samples were prepared by mixing different molar ratios of fibers (**1a**)_6_ to the mixture of trimers and tetramers (**1a**)_3_/(**1a**)_4_ prepared from **1a**. As described above, fibers of (**1a**)_6_ were prepared from monomers of building block **1a** (500 μL, 2.0 mM) upon partial oxidation (50%) using sodium perborate (12.5 μL, 40 mM) in borate buffer (50 mM, pH 8.2). The sample was thoroughly mixed using a micropipet, and the vial was subsequently closed with a Teflon septum screw cap. The mixture was then placed in a metal HPLC vial holder and stirred at 1200 rpm at 45 °C for 4 days. To prepare a mixture of trimers and tetramers (**1a**)_3_/(**1a**)_4_, building block **1a** (500 μL, 2.0 mM) was fully oxidized using sodium perborate (25 μL, 40 mM) in borate buffer (50 mM in boron atoms, pH 8.2). The sample was thoroughly mixed using a micropipet and stored at room temperature for around 1 h.

To test the ability of the sensor to track the emergence of replicators, we prepared libraries having different ratios (shown in Table 1) of trimers–tetramers (**1a**)_3_/(**1a**)_4_ and fibers (**1a**)_6_, which represent different stages of replicator emergence. Fluorescence patterns were recorded following excitation at 440 and 530 nm, respectively. Principle component analysis was applied to discriminate among the emission patterns obtained at the following excitation and emission wavelengths: λ_ex_: 440 nm, λ_em_: 490, 540, 570, 595, 640, 675, 700 nm; and λ_ex_: 530 nm, λ_em_: 570, 595, 640, 675, 700 nm (Figure S7).

### Real-Time Study of the Emergence of Replicators Based on Building Block **1a** Using ID-Probe **2a**

Building block **1a** (stored at −20 °C) was added (around 1 mg) to an HPLC vial (12 × 32 mm), and around 0.5 mL of freshly prepared borate buffer (50 mM in boron atoms, pH 8.2) was added. The pH of the solution was measured and, if necessary, adjusted using 1.0 M NaOH solution. The final concentration of the stock solution was 2.0 mM. Then 7.5 μL of this stock solution and 2.0 μL of a solution of **2a** (0.5 mM in DMSO) were individually transferred to an HPLC vial (12 × 32 mm) containing a Teflon-coated magnetic stirring bar (5 × 2 mm, VWR) to obtain the desired concentration of **1a** (30 μM in units of building block) and **2a** (2.0 μM) in borate buffer (50 mM, pH 8.2; final DMSO concentration <0.5%) (vial A).

For the control experiments, 7.5 μL of stock solution of **1a** (2.0 mM) and 2.0 μL of fresh DMSO solution were transferred to an HPLC vial (12 × 32 mm) containing a Teflon-coated magnetic stirring bar (5 × 2 mm, VWR) to obtain the desired concentration of **1a** (30 μM in units of building block) in borate buffer (50 mM, pH 8.2; final DMSO concentration <0.5%) (vial B).

For the second control experiment, 2.0 μL of **2a** (0.5 mM in DMSO) was transferred to an HPLC vial (12 × 32 mm) containing a Teflon-coated magnetic stirring bar (5 × 2 mm, VWR) to obtain the desired concentration of **2a** (2 μM) in borate buffer (50 mM, pH 8.2) solution (final DMSO concentration <0.5%) (vial C).

After initial fluorescence measurements, UPLC and LC-MS measurement vials were subsequently closed with Teflon septum screw caps, placed in a metal HPLC vial holder, and stirred at 1200 rpm at 30 °C in the dark. Fluorescence readings from the mixture of **1a** and **2a** at various time points were recorded using excitation at 440 and 530 nm, respectively. At the same time, the composition of the mixture was periodically analyzed by UPLC and the formation of fibers was analyzed by TEM measurements. All these experiments were repeated four times on independent days using freshly prepared samples starting from the weighing step. Principle component analysis was applied to discriminate among the emission patterns obtained at various time points using the following excitation and emission wavelengths: λ_ex_: 440 nm, λ_em_: 490, 540, 570, 595, 640, 675, 700 nm; and λ_ex_: 530 nm, λ_em_: 570, 595, 640, 675, 700 nm (Figures 5b, S9).

### Transmission Electron Microscopy

To confirm the formation of replicator fibers from different building blocks as described above, samples (5.0 μL) were applied to carbon-coated copper TEM grids (400 mesh) for 40 s at room temperature. Excess liquid was removed with filter paper, and the grids were negatively stained with saturated uranyl acetate (5.0 μL), blotted on filter paper after incubation for 20 s, and air-dried. TEM was recorded on a Philips CM120 electron microscope operating at 120 kV. TEM images (Figures 5b, S3b, S14b, S15b, and S16b) were recorded on a slow-scan charge-coupled device camera (Gatan).

### Ultraperformance Liquid Chromatography Analysis

UPLC analyses were performed on a Waters Acquity UPLC H-class system equipped with a PDA detector. A reversed-phase UPLC column (Aeris 1.7 μm XB-C18 150 × 2.10 mm, purchased from Phenomenex) was used for the analyses of all samples, while the UV absorbance was monitored at 254 nm. Note that the different sized macrocycles formed from the different building blocks have comparable extinction coefficients at 254 nm. The column temperature was equilibrated at 30 °C prior to injections. The elution phases consisted of UPLC grade water with 0.1% TFA (eluent A) and acetonitrile with 0.1% TFA (eluent B) at a constant flow rate of 0.3 mL min^–1^. All UPLC samples were injected using the solvent gradients shown in Table 2. For the analysis of the samples, peaks were assigned (using LC-MS data), then integrated, and the resulting peak areas were used to construct the kinetics plots.

**Table 2:** Gradient Used for UPLC Analysis

time (min)	eluent A (%)	eluent B (%)
0.00	90	10
1.00	90	10
1.30	75	25
3.00	72	28
11.00	60	40
11.50	5	95
12.00	5	95
12.50	90	10
17.00	90	10

## Supporting Information Available

Synthesis of **2a**–**e**, Figures S1–S18, and Tables S1–S4 (PDF)
