# Supplementary material for: An Optical Probe for Real-Time Monitoring of Self-Replicator Emergence and Distinguishing between Replicators
Source: J Am Chem Soc. 2022 Feb 9;144(7):3074–82. doi: 10.1021/jacs.1c11594 (PMC8874894; doi:10.1021/jacs.1c11594)
Supplement: Supplementary file 1 — ja1c11594_si_001.pdf [file ja1c11594_si_001.pdf]

An Optical Probe for Real-Time Monitoring of Self-Replicator Emergence and Distinguishing between Replicators

Joydev Hatai,[1] Yigit Altay,[1] Ankush Sood,[1] Armin Kiani,[1] Marcel J. Eleveld,[1] Leila Motiei,[2] David Margulies*,[2] and Sijbren Otto*[1]

[1]Centre for Systems Chemistry Stratingh Institute, University of Groningen, Nijenborgh 4, 9747 AG Groningen, The Netherlands

[2]Department of Organic Chemistry, Weizmann Institute of Science, Rehovot 7610001, Israel

Table of Contents

Materials and Methods	S3
Abbreviations	S3
Synthetic Procedures	S4
Synthesis of 3,5-dimercaptobenzoyl group containing peptide **1a-d**	S4
Synthesis of compound **2b**	S5
Synthesis of compound **2d** and **21**	S8
Synthesis of compound **2a**	S12
Synthesis of compound **2c**	S14
Synthesis of compound **2e**	S16
Figures and Tables	S19
References	S33

Materials and Methods

All solvents and reagents were obtained from commercial suppliers and used without further purification, unless otherwise noted. N-Boc-*cis*-4-N-Fmoc-amino-L-proline and Fmoc-Lys(Boc)-OH were purchased from Chem-Impex International (Wood Dale, IL). H_2N-PEG_4-tBu and sulfo-Cy5 NHS ester were purchased from Broadpharm, Inc. (San Diego, CA), and Lumiprobe Corporation, respectively. Dry solvents were purchased from Sigma Aldrich. Ultra-performance liquid chromatography (UPLC) analyses were performed on a Waters Acquity UPLC H-class system equipped with a PDA detector. A reversed-phase UPLC column (Aeris 1.7 μm XB-C18 150 × 2.10 mm, purchased from Phenomenex) was used in the analyses of all samples, while UV absorbance was monitored at 254 nm. The column temperature was equilibrated at 30 ^{o}C prior to injections. The elution phases consisted of UPLC grade ACN with 0.1% TFA (eluent A) and water with 0.1% TFA (eluent B) at a flow rate of 0.3 ml min^{-1}. UPLC–mass spectrometry experiments were performed by direct injection of samples using a Waters Acquity UPLC-H-class system coupled to a Waters Xevo-G2 TOF. The mass spectrometer was operated at positive electrospray ionization mode and under the following conditions: capillary voltage: 2.5 kV; sampling cone voltage: 30 V; extraction cone voltage: 4.0 V. Source and desolvation temperatures were set at 140 °C and 500 °C. The ^{1}H NMR and ^{13}C NMR spectra were recorded on a Bruker Advance 400 MHz or a Bruker Advance 600 MHz spectrometer. The chemical shifts are represented in ppm on the δ scale down field from TMS as the internal standard. The following abbreviations were used to describe the peaks: br-broad, s-singlet, d-doublet, t-triplet, q-quartet, and m-multiplet. Preparative HPLC was carried out on an Shimadzu Prominence purification system, equipped with an autosampler, a UV-Vis dual wavelength detector, and fraction collector using XSelect CSH C18 5μm 10x250 preparative OBD column and operated using Shimadzu LC Solutions software. The elution phases consisted of 10% ACN in H_2O with 0.1% TFA (eluent A) and 90% acetonitrile in H_2O with 0.1% TFA (eluent B).

Abbreviations. Acetonitrile (ACN), dichloromethane (DCM), N,N'-diisopropylethylamine(DIPEA), N,N'-dimethylformamide (DMF), dimethyl sulfoxide (DMSO), 1-[bis(dimethylamino)methylene]-1H-1,2,3-triazolo[4,5-b]pyridinium3-oxid hexafluorophosphate (HATU), Principle component analysis (PCA), methanol (MeOH), Diethyl ether (Et_2O), reverse phase high-performance liquid chromatography (RP-HPLC), Ultra-Performance Liquid Chromatography (UPLC), thioflavin T (ThT), Sulforhodamine B (SRB), sulfo-Cy5 (sCy5), transmission electron microscopy (TEM), trifluoroacetic acid (TFA), .

Synthetic Procedures

Synthesis of 3,5-dimercaptobenzoyl group containing peptide 1a-d

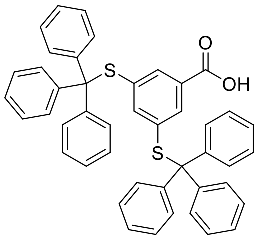

3,5-bis(tritylthio)-benzoic acid

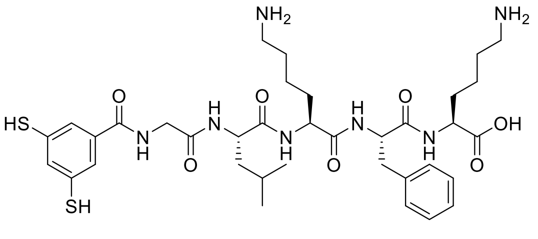

1a

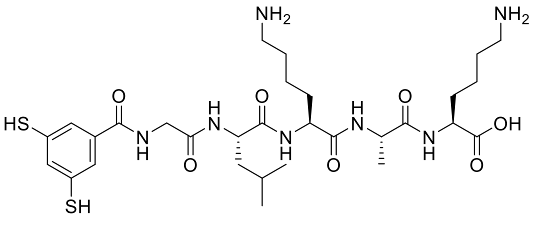

1b

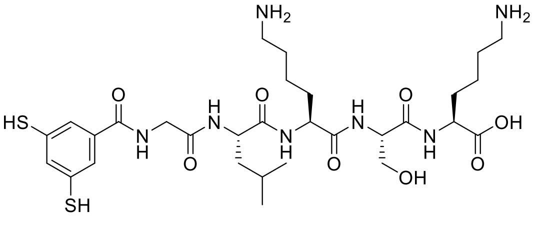

1c

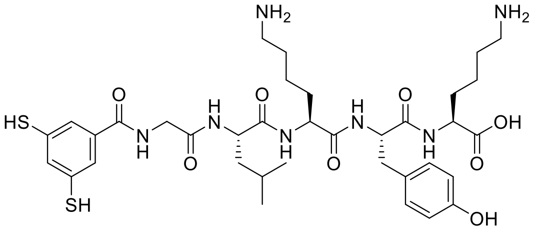

1d

The core building block 3,5-bis(tritylthio)-benzoic acid was synthesized based on a previously reported procedure.[1] Peptides **1a-d** were obtained from Cambridge Peptides Ltd. by coupling 3,5-bis(tritylthio)-benzoic acid at the N-termini.

Synthesis of compound 2b (overview)

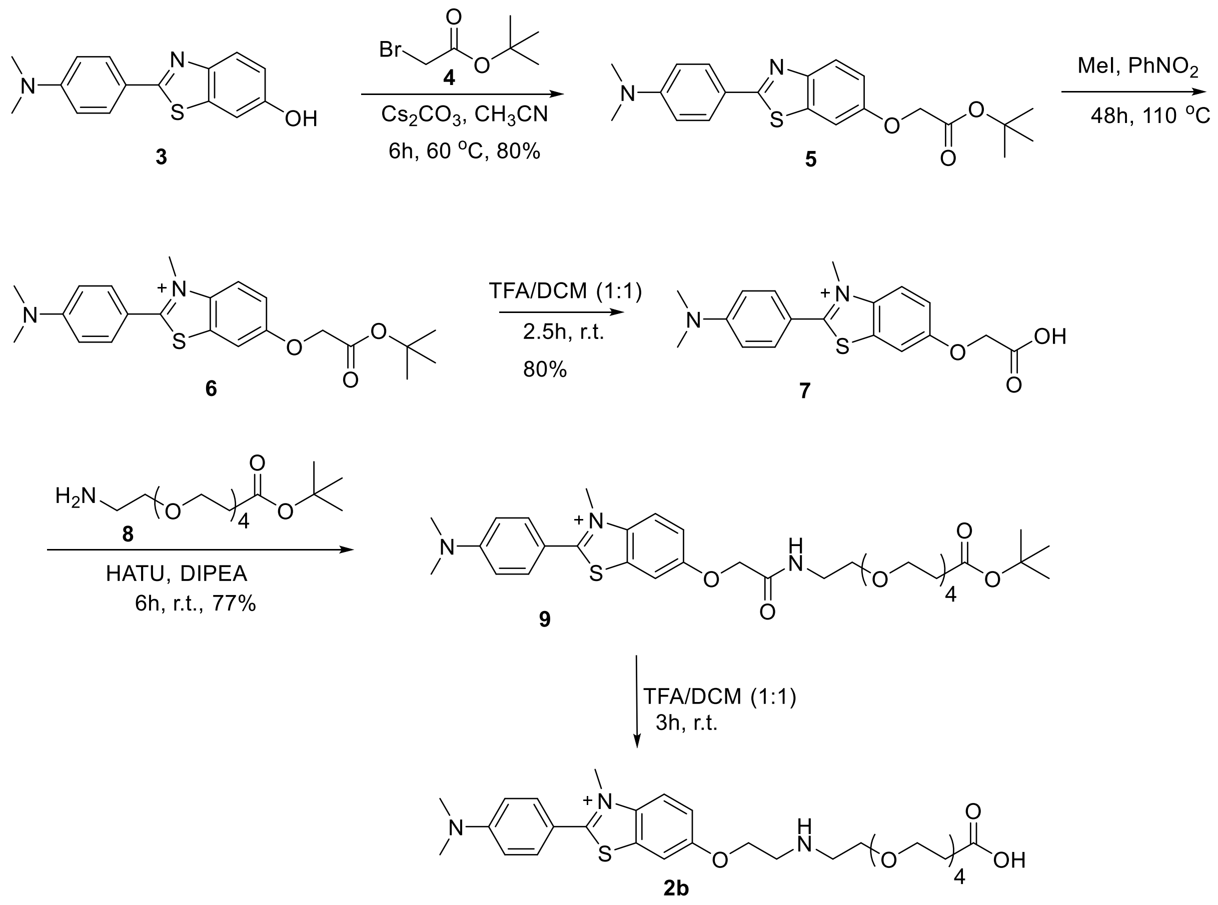

Compound **3** was synthesized starting from commercially available building blocks based on a reported procedure.[2]

Synthesis of compound 5:

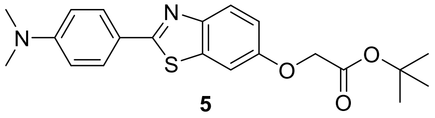

5

This compound was synthesized by slight modification of a reported procedure.[3] A mixture of compound **3** (0.10 g, 0.37 mmol) and Cs_2CO_3 (0.35 g, 2.20 mmol) in anhydrous CH_3CN (3.0 mL) was stirred for 30 min at room temperature. Then compound **4** (0.43 g, 0.33 mL, and 2.20 mmol) was added and the reaction mixture was refluxed for 5 h at 65 °C. After the reaction was completed (judging by silica TLC Plate, 20% ethyl acetate in hexane, R_f: 0.1 (compound **3**), 0.5 (product)), it was filtered and the solvent was evaporated under reduced pressure. The white residue was washed with ethyl acetate (3 × 10.0 mL) to afford a white solid **5** (0.114 g) in a 80% yield. ^{1}H NMR (400 MHz, $CDCl_3$): δ 7.91-7.85 (m, 3H), 7.29 (d, J = 3.4 Hz, 1H), 7.05 (dd, J_1 = 11.9 Hz, J_2 = 3.4 Hz, 1H), 6.71 (d, J = 11.9 Hz, 2H), 4.57 (s, 2H), 3.02 (s, 6H), 1.49 (s, 9H). ^{13}C NMR (100 MHz, $CDCl_3$): δ 168.0, 167.1, 155.5, 152.0, 149.5, 135.7, 128.7, 122.8, 121.5, 115.3, 111.8, 105.9, 82.6, 66.5, 40.2, 28.1. HRMS-ESI (*m/z*): $[M+H]^+$ calcd. for $C_{21}H_{25}N_2O_3S^+$ 385.1580; found 385.1573.

Synthesis of compound 7:

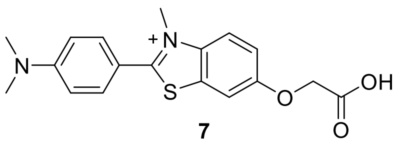

7

Compound **5** (0.20 g, 0.52 mmol), MeI (1.48 g, 0.64 mL, 10.40 mmol) and nitrobenzene (8.0 mL) were mixed in a pressure vial and stirred for 48 h at 110 °C. The mixture was cooled down to room temperature and poured in hexane (150.0 mL) with continuous stirring. The precipitate was filtered and washed with hexane (3 × 30.0 mL) to obtain **6** as a yellowish solid which was used in the next step without further purification. To a solution of **6** (0.10 g, 0.25 mmol) in DCM (2.0 mL) under ice cooling was slowly added trifluoroacetic acid (2.0 mL). The reaction mixture was allowed to warm up to room temperature and stirring was continued for another 2.5 h. After the reaction was completed, DCM and TFA were evaporated. The traces of TFA were removed by co-evaporation with DCM. The solid residue was washed with DCM (3 × 20.0 mL) to yield **7** (0.069 g) as a yellow solid in 80% yield. ^{1}H NMR (400 MHz, $(CD_3)_2SO$): δ 13.19 (br, 1H), 8.15 (d, J = 9.3 Hz, 1H), 7.96 (d, J = 2.6 Hz, 1H), 7.78 (d, J = 8.7 Hz, 2H), 7.49 (dd, J_1 = 9.3 Hz, J_2 = 2.6 Hz, 1H), 6.96 (d, J = 8.7 Hz, 2H), 4.86 (s, 2H), 4.21 (s, 3H), 3.10 (s, 6H). ^{13}C NMR (100 MHz, $(CD_3)_2SO$): δ 171.5, 169.5, 157.2, 154.4, 137.2, 132.0, 129.5, 118.2, 117.8, 111.9, 110.9, 107.6, 65.0, 39.7, 38.3. HRMS-ESI (*m/z*): $[M]^+$ calcd. for $C_{18}H_{19}N_2O_3S^+$ 343.1110; found 343.1104.

Synthesis of compound 2b:

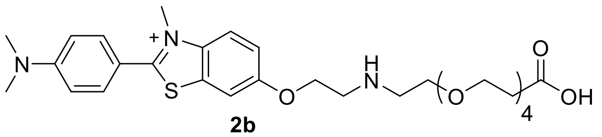

2b

Compound **7** (0.10 g, 0.29 mmol) was dissolved in DCM (5.0 mL) and the solution was basified (judging by wet pH paper) with DIPEA (0.10 mL, 0.58 mmol). Coupling reagent HATU (0.22 g, 0.58 mmol) was added to the above reaction mixture and stirred for 10 min at room temperature. Then **8** (0.09 g, 0.32 mmol) was added and the reaction was continued at room temperature for another 3h under argon. After the reaction was completed (judging by silica TLC Plate, 4% methanol in DCM, R_f: 0.2 (compound **7**), 0.6 (product)), the solvent was removed in vacuum and the residue was dissolved in DCM (10.0 mL) and washed twice with water (20.0 mL). The organic layer was dried over anhydrous sodium sulfate and evaporated to dryness. The crude product was purified by column chromatography (silica gel, 1.5% MeOH in DCM) to afford **9** as a yellow foamy substance (0.145 g, 77%). ^{1}H NMR (400 MHz, $CDCl_3$): δ 7.84 (d, J = 9.3 Hz, 1H), 7.63 (d, J = 9.0 Hz, 2H), 7.51 (d, J = 2.5 Hz, 1H), 7.37 (dd, J_1 = 9.0 Hz, J_2 = 2.5 Hz, 1H), 7.23 (t, J = 5.7 Hz, 1H), 6.82 (d, J = 9.0 Hz, 2H), 4.52 (s, 2H), 4.21 (s, 3H), 3.65 (t, J = 6.5 Hz, 1H), 3.60-3.51 (m, 14H), 3.50 (q, J = 5.3 Hz, 2H), 3.09 (s, 6H), 2.45 (t, J = 6.5 Hz, 2H), 1.40 (s, 9H). ^{13}C NMR (100 MHz, $CDCl_3$): δ 174.2, 173.1, 169.3, 159.5, 156.0, 139.6, 134.2, 131.7, 121.5, 119.7, 114.6, 113.0, 109.1, 82.7, 72.5, 72.4, 72.3, 72.2, 71.6, 69.8, 68.9, 42.2, 41.0, 40.3, 38.3, 30.2. FT-IR: [cm^{-1}]: 2914, 2871, 1722, 1674, 1599, 1541, 1508, 1479, 1441, 1381, 1270, 1232, 1202, 1104, 1071, 941. HRMS-ESI (*m/z*): $[M]^+$ calcd. for $C_{33}H_{48}N_3O_8S^+$ 646.3156; found 646.3141. To obtain compound **2b**, the tert-butyl ester group of **9** was deprotected by adding TFA (2.0 mL) to a solution of **9** (0.10 g, 0.17 mmol) in DCM (2.0 mL) under ice cooling condition. The reaction mixture was allowed to warm up to room temperature and stirring was continued for another 3 h. After the reaction was completed, DCM and TFA were evaporated. The traces of TFA were removed by co-evaporation with DCM and the product was used in the next step without further purification.

Synthesis of compound 2d and 21 (overview)

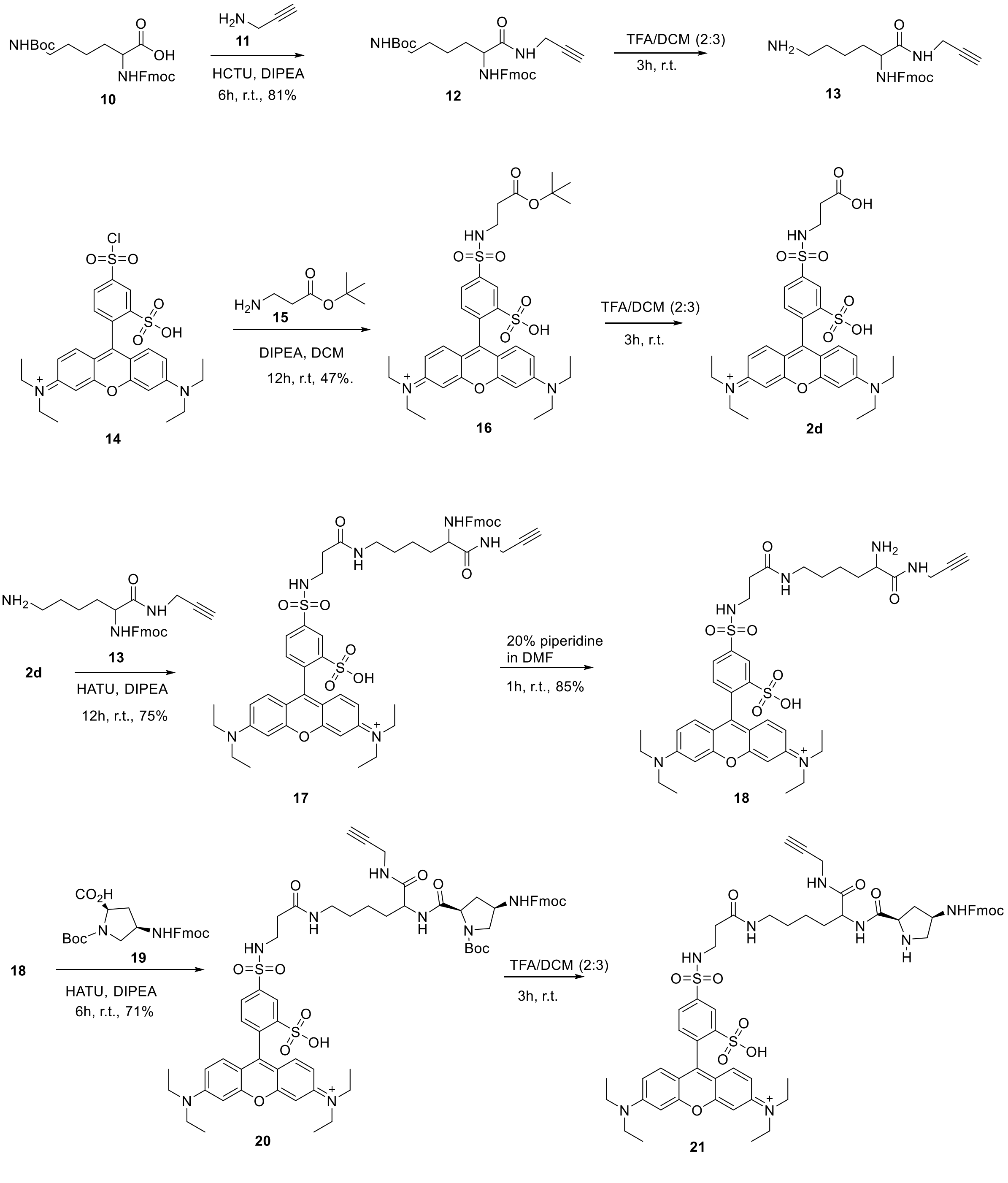

Synthesis of compound 13:

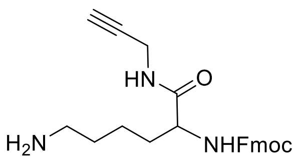

13

Compound **10** (0.10 g, 0.21 mmol) and HATU (0.16 g, 0.43 mmol) were dissolved in DCM (4.0 mL); the solution was basified (judging by wet pH paper) with DIPEA (0.04 mL, 0.43 mmol) and stirred at room temperature for 10 min. Then propargylamine (**11**) (15 µL, 0.24 mmol) was added and the reaction mixture was stirred at room temperature for 4 h under argon. The solvent was removed in vacuum and the residue was suspended in water (50.0 mL) and stirred for 4h followed by vacuum filtration, and dried under reduced pressure. The crude material washed with hexane and purified by column chromatography (silica gel, 75% EtOAc in hexane) to afford **12** as a white solid (0.087 g, 81%). ^{1}H NMR (400 MHz, $CDCl_3$): δ 7.75 (d, *J* = 7.5 Hz, 2H), 7.59 (d, *J* = 7.5 Hz, 2H), 7.39 (t, *J* = 7.5 Hz, 2H), 7.30 (t, *J* = 7.5 Hz, 2H), 7.12 (br s, 1H), 5.90 (d, *J* = 8.3, 1H), 4.93 (br s, 1H), 4.50 (d, *J* = 5.7 Hz, 2H), 4.39 (br s, 1H), 4.27 (t, *J* = 7.2 Hz, 2H), 4.02 (br s, 3H), 3.09 (br s, 3H), 1.97-1.7 (m, 2H), 1.57-1.35 (br m, 13H); ^{13}C NMR (100 MHz, $CDCl_3$): δ 171.8, 156.4, 143.8, 141.3, 127.8, 127.1, 125.1, 120.0, 79.4, 71.7, 67.2, 54.6, 53.5, 47.1, 40.0, 32.2, 29.6, 29.2, 28.5, 22.5. HRMS-ESI (m/z): $[M+H]^+$ calcd. for $C_{29}H_{36}N_3O_5^+$ 506.2649; found 506.2641. To obtain compound **13**, trifluoroacetic acid (2.0 mL) was added to a solution of compound **12** (0.10 g, 0.20 mmol) in DCM (2.0 mL) under ice cooling condition. Then the reaction mixture was allowed to warm up to room temperature and stirring was continued for another 3 h. After the reaction was completed, DCM and TFA were evaporated. The traces of TFA were removed by co-evaporation with DCM five times and the residue was dried under high vacuum overnight and used in the next step without further purification.

Synthesis of compound 2d:

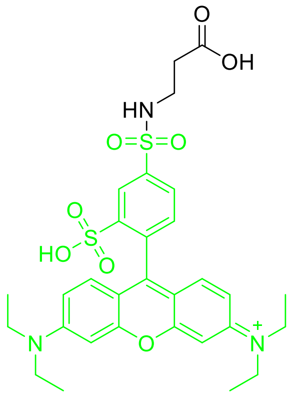

2d

Compound **14** (3.18 g, 5.50 mmol) was added to a mixture containing **15** (1.00 g, 5.50 mmol) and DIPEA (1.92 mL, 11.04 mmol) in anhydrous DCM (30.0 mL) under ice cooling. The reaction mixture was monitored by silica TLC plate (10% methanol in DCM, R_f: 0.1 (compound **14**), 0.6 (product)). After the reaction was completed (6h), the solvent was removed in vacuum and the residue was washed with hexane. The residue was dissolved in DCM (40.0 mL) and washed twice with 5% HCl (20.0 mL) and water (30.0 mL). The organic layer was dried over anhydrous magnesium sulfate, evaporated to dryness, and the crude material was purified by column chromatography (silica gel, 10% MeOH in DCM) to afford **16** as a deep brown solid (1.77 g, 47%). ^{1}H NMR (400 MHz, $(CD_3)_2SO$): δ 8.41 (s, 1H), 8.03 (br s, 1H), 7.93 (d, *J* = 8.0, 1H), 7.48 (d, *J* = 8.0, 1H), 7.01 (d, *J* = 9.2, 2H), 7.01 (d, *J* = 9.2, 2H), 6.99 (t, *J* = 9.2 Hz, 2H), 6.94 (s, 2H), 3.70-3.59 (br m, 8H), 3.05 (t, *J* = 6.5, 2H), 2.42 (t, *J* = 6.5, 2H), 1.41 (s, 9H), 1.20 (t, *J* = 7.0, 12H). ^{13}C NMR (100 MHz, $(CD_3)_2SO$): δ 169.9,

157.5, 157.1, 155.0, 148.0, 141.2, 133.1, 132.7, 126.6, 125.7, 113.9, 95.4, 80.2, 45.7, 38.6, 35.3, 27.8, 12.5. HRMS-ESI (m/z): $[M]^+$ calcd. for $C_{34}H_{44}N_3O_8S_2^+$ 686.2564; found 686.2563. To obtain compound **2d**, trifluoroacetic acid (2.0 mL) was added to a solution of compound **16** (0.10 g, 0.15 mmol) in DCM (4.0 mL) under ice cooling. Then the reaction mixture was allowed to warm up to room temperature and stirring was continued for another 4 h. After the reaction was completed, DCM and TFA were evaporated. The traces of TFA were removed by co-evaporation with DCM five times and the residue was washed with Et_2O and dried under reduced pressure and used in the next step without further purification.

Synthesis of compound 17:

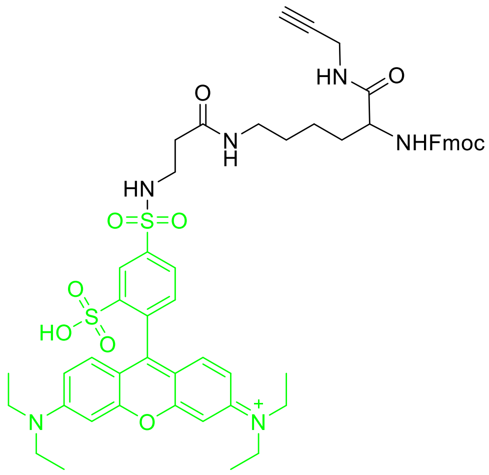

17

Compound **2d** (0.10 g, 0.16 mmol) was dissolved in DCM (4.0 mL) and the solution was basified (judging by wet pH paper) with DIPEA (69 µL, 0.40 mmol). Coupling reagent HATU (0.12 g, 0.32 mmol) was added to the above reaction mixture which was stirred for 10 min at room temperature. Then compound **13** (0.07 g, 0.16 mmol) was added and reaction was continued at room temperature for another 6h under argon. After the reaction was completed (judging by silica TLC plate, 6% methanol in DCM, R_f: 0.1 (compound **2d**), 0.5 (product)), the solvent was removed in vacuum and the residue was suspended in H_2O (50.0 mL) and stirred for 6h followed by vacuum filtration, washed with hexane and dried under reduced pressure. The crude product was purified by column chromatography (silica gel, 7% MeOH in DCM) to afford **17** as a deep brown solid (0.121 g, 75%). ^1H NMR (400 MHz, $CDCl_3$): δ 8.75 (s, 1H), 7.68 (d, J = 7.5 Hz, 2H), 7.55 (d, J = 6.5 Hz, 2H), 7.48 (br s, 1H), 7.31 (t, J = 7.5 Hz, 2H), 7.25-7.16 (m, 5H), 6.81 (d, J = 9.2 Hz, 2H), 6.7 (br s, 1H), 6.63 (s, 2H), 4.24 (d, J = 7.0 Hz, 1H), 4.17-4.01 (m, 2H), 3.86 (br s, 2H), 3.49 (br s, 8H), 3.28 (br s, 2H), 3.09 (br s, 2H), 2.61 (br s, 2H), 2.32 (t, J = 6.8 Hz, 2H), 2.12 (s, 1H), 1.78-1.56 (m, 2H), 1.44-1.28 (m, 4H), 1.23 (br m, 12H); ^{13}C NMR (100 MHz, $CDCl_3$): δ 172.4, 171.5, 157.8, 157.7, 156.4, 155.5, 146.7, 144.0, 142.3, 141.2, 133.7, 133.3, 130.5, 127.9, 127.7, 127.2, 127.1, 125.5, 119.8, 114.2, 113.0, 95.7, 80.0, 77.4, 71.2, 66.9, 54.9, 54.2, 47.1, 45.9, 42.2, 40.2, 38.8, 35.4, 32.2, 29.7, 28.9, 28.4, 22.5, 18.5, 17.2, 12.7. HRMS-ESI (m/z): $[M]^+$ calcd. for $C_{54}H_{61}N_6O_{10}S_2^+$ 1017.3881; found 1017.3885.

Synthesis of compound 18:

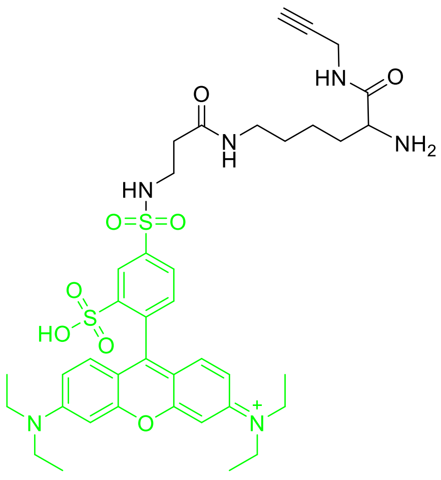

18

Compound **17** (0.10 g, 0.10 mmol) was dissolved in 20% piperidine in DMF (3.0 mL). The reaction mixture was stirred for 3 h at room temperature in the dark. After the reaction was completed (judging by silica TLC plate, 8% methanol in DCM, R_f: 0.7 (compound **17**), 0.2 (product)), excess H_2O (40.0 mL) was added and the mixture was stirred for 1h. Then the mixture was extracted with DCM (20.0 mL) and washed with brine (20.0 mL), and again with water (2 × 20.0 mL). The organic layer was dried over anhydrous sodium sulfate and evaporated to dryness to obtain crude **18**. The crude product was purified by column chromatography (silica gel, 20% MeOH in DCM) to afford **18** as a deep brown solid (0.066 g, 85%). ^{1}H NMR (400 MHz, $CDCl_3$): δ 8.59 (d, J = 1.9 Hz, 1H), 8.06 (dd, J_1 = 8.8 Hz, J_2 = 1.9 Hz, 1H), 7.47 (d, J = 8.8 Hz, 1H), 7.08 (d, J = 9.5 Hz, 2H), 6.95 (dd, J_1 = 9.5 Hz, J_2 = 2.5 Hz, 2H), 6.88 (d, J = 2.5 Hz, 2H), 3.93-3.91 (m, 2H), 3.62 (q, J = 7.0 Hz, 8H), 3.32 (d, J = 7.0 Hz, 1H), 3.26 (p, J = 1.7 Hz, 2H), 3.22 (t, J = 6.8 Hz, 2H), 3.14 (t, J = 6.8 Hz, 2H), 2.54 (t, J = 2.5 Hz, 2H), 2.4 (t, J = 6.8 Hz, 2H), 1.69-1.34 (m, 5H), 1.25 (t, J = 7.0 Hz, 12H), 1.12 (t, J = 7.0 Hz, 1H); ^{13}C NMR (100 MHz, $CDCl_3$): δ 176.5, 172.8, 159.2, 157.7, 156.9, 147.1, 143.5, 135.3, 133.6, 132.3, 129.2, 127.6, 115.1, 114.9, 96.9, 80.4, 79.4, 79.0, 78.7, 72.2, 55.6, 49.4, 46.7, 40.6, 40.0, 37.3, 35.5, 29.9, 29.4, 23.7, 12.8. HRMS-ESI (m/z): $[M]^+$ calcd. for $C_{39}H_{51}N_6O_8S_2^+$ 795.3204; found 795.3206.

Synthesis of compound 21:

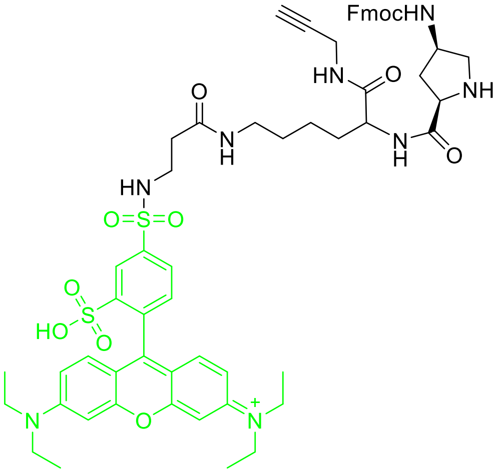

20

Compound **19** (0.05 g, 0.11 mmol) was dissolved in DCM (5.0 mL) and the solution was basified (judging by wet pH paper) with DIPEA (44 µL, 0.25 mmol). Coupling reagent HATU (0.10 g, 0.25 mmol) was added to the above reaction mixture which was stirred for 10 min at room temperature. Then compound **18** (0.10 g, 0.13 mmol) was added and reaction was continued at room temperature for another 24h under argon. After the reaction was completed (judging by silica TLC plate, 8% methanol in DCM, R_f: 0.9 (compound **19**), 0.7 (product)), the solvent was removed in vacuum and the residue was dissolved in DCM (10.0 mL) and washed with water (20.0 mL), brine (20.0 mL), and again with water (2 × 20.0 mL). The organic layer was dried over anhydrous sodium sulfate and evaporated to dryness. The crude product was purified by column

chromatography (silica gel, 7% MeOH in DCM) to afford **20** as a deep brown solid (0.109 g, 71%). ^{1}H NMR (400 MHz, $CDCl_3$): δ 8.73 (s, 1H), 8.02 (d, *J* = 6.9 Hz, 1H), 7.68 (d, *J* = 6.9 Hz, 3H), 7.56 (q, *J* = 7.0 Hz, 3H), 7.40 (br s, 1H), 7.31 (t, *J* = 7.4 Hz, 4H), 7.28-7.17 (br m, 3H), 7.07 (br s, 1H), 6.84 (q, *J* = 9.0 Hz, 3H), 6.63 (br d, 2H), 4.42-4.06 (m, 6H), 3.85 (br s, 2H), 3.65-3.40 (br m, 10H), 3.33 (s, 2H), 3.08 (br d, 2H), 2.55 (br s, 2H), 2.37 (br s, 2H), 2.07 (s, 1H), 2.05-1.91 (m, 1H), 1.72 (br s, 1H), 1.45-1.20 (m, 23H); ^{13}C NMR (100 MHz, $CDCl_3$): δ 173.0, 171.9, 171.3, 157.9, 157.8, 156.2, 155.5, 155.3, 146.9, 144.0, 144.0, 142.3, 141.1, 133.6, 133.2, 130.3, 127.8, 127.6, 127.2, 126.9, 125.5, 119.8, 114.2, 113.8, 95.7, 80.7, 80.1, 77.4, 70.8, 66.8, 59.8, 53.6, 50.2, 47.1, 45.9, 40.2, 38.8, 35.6, 30.9, 28.9, 28.4, 22.3, 12.6. FT-IR: [cm^{-1}]: 3379, 2922, 1652, 1589, 1530, 1415, 1336, 1274, 1246, 1180, 1134, 1075, 1028, 977, 921. HRMS-ESI (*m/z*): $[M]^+$ calcd. for $C_{64}H_{77}N_8O_{13}S_2^+$ 1229.5046; found 1229.5039. To obtain compound **21**, trifluoroacetic acid (2.0 mL) was added to a solution of compound **21** (0.10 g, 0.08 mmol) in DCM (4.0 mL) under ice cooling. Then the reaction mixture was allowed to warm up to room temperature and stirring was continued for another 3.5 h. After the reaction was completed, DCM and TFA were evaporated and excess diethyl ether (25.0 mL) was added and the suspension was filtered. The residue was dried under high vacuum for 5h and used in the next step without further purification.

Synthesis of compound 2a (overview)

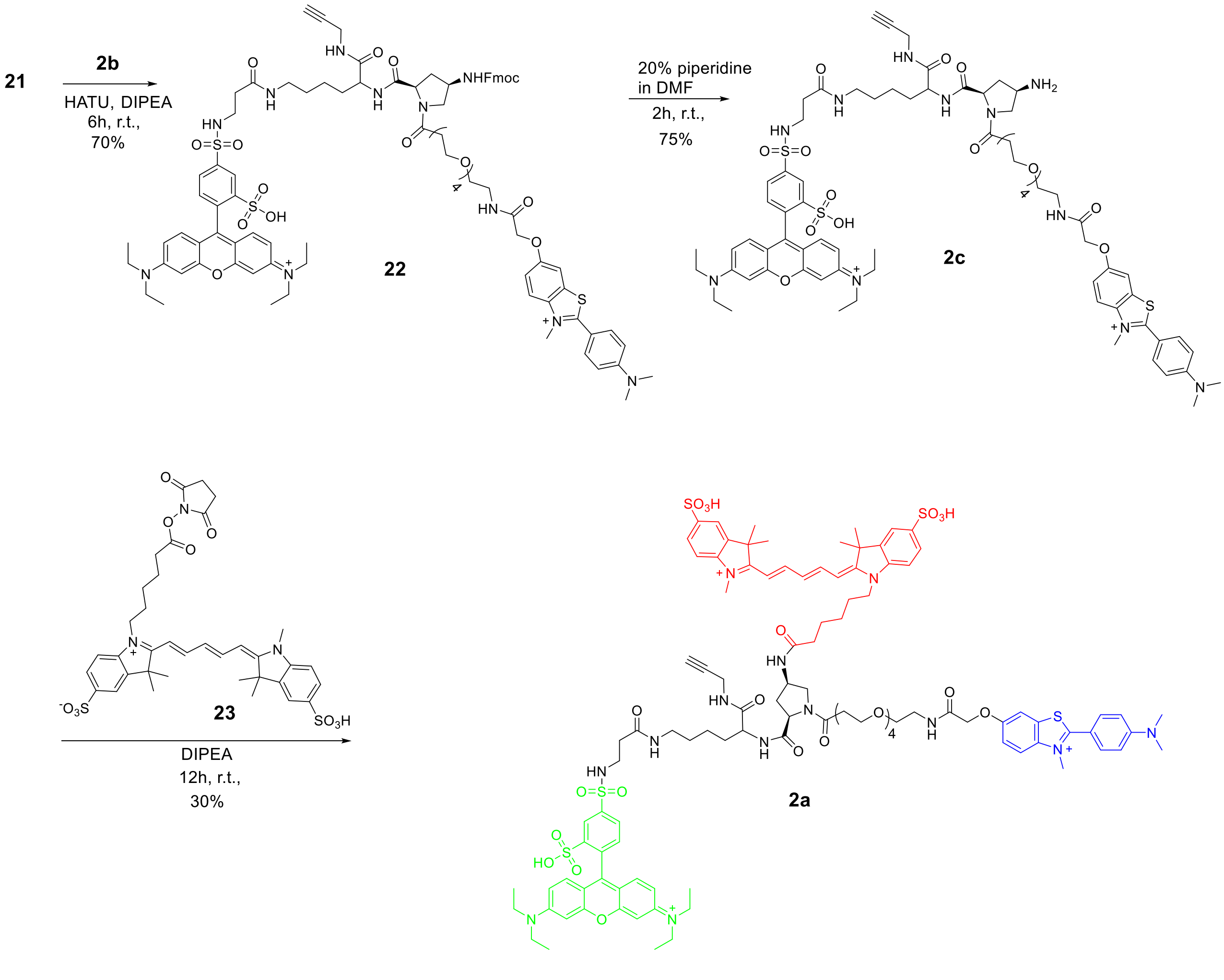

Synthesis of compound 22:

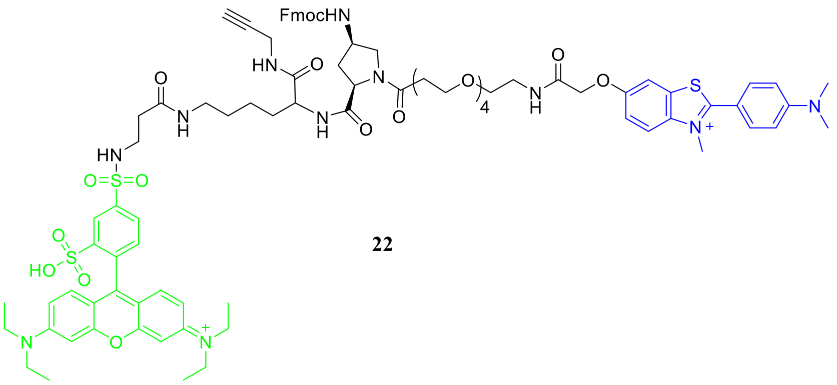

22

Compound **2b** (0.05 g, 0.09 mmol) was dissolved in DCM (5.0 mL) and the solution was basified (judging by wet pH paper) with DIPEA (30 µL, 0.18 mmol). Coupling reagent HATU (0.07 g, 0.18 mmol) was added to the above reaction mixture which was stirred for 10 min at room temperature. Then compound **21** (0.10 g, 0.09 mmol) was added and reaction was continued at room temperature for another 6h under argon. After the reaction was completed (judging by silica TLC plate, 8% methanol in DCM, R_f: 0.1 (compound **2b**), 0.2 (compound **21**), 0.7 (product)), the solvent was removed in vacuum and the residue was dissolved in DCM (10.0 mL) and washed with water (20.0 mL), brine (20.0 mL), and again with water (2 × 20.0 mL). The organic layer was dried over anhydrous sodium sulfate and evaporated to dryness. The crude product was purified by column chromatography (silica gel, 12% MeOH in DCM) to afford **22** (0.105 g, 70%). ^{1}H NMR (400 MHz, $CDCl_3$): δ 8.58 (s, 1H), 8.40 (br s, 1H), 8.12-8.04 (m, 3H), 7.95 (br s, 1H), 7.80 (t, J = 5.3 Hz, 1H), 7.70-7.64 (m, 5H), 7.60-7.48 (m, 5H), 7.30 (t, J = 7.7 Hz, 2H), 7.26-7.14 (m, 5H), 6.83-6.75 (m, 4H), 6.66 (s, 2H), 4.63 (s, 2H), 4.56 (t, J = 8.1 Hz, 1H), 4.36 (br s, 4H), 4.17 (d, J = 7.7 Hz, 2H), 4.07 (q, J = 7.6 Hz, 1H), 3.97-3.85 (m, 2H), 3.77 (q, J = 3.5 Hz, 2H), 3.6 (s, 8H), 3.58 (s, 6H), 3.54-3.42 (m, 8H), 3.21 (br s, 4H), 3.09 (s, 6H), 3.02-2.94 (br m, 3H), 2.80-2.61 (m, 2H), 2.58-2.45 (m, 2H), 2.24-2.20 (m, 1H), 2.14 (t, J = 2.5 Hz, 1H), 1.83-1.75 (m, 2H), 1.63-1.36 (m, 4H), 1.25 (t, J = 6.7 Hz, 12H); ^{13}C NMR (100 MHz, $CDCl_3$): δ 175.2, 174.8, 174.4, 170.2, 161.4, 160.4, 160.3, 158.9, 158.1, 156.3, 149.9, 146.6, 146.7, 140.0, 143.6, 140.2, 135.9, 134.9, 132.7, 132.4, 130.2, 129.8, 129.7, 129.6, 128.2, 122.4, 120.7, 116.9, 116.3, 115.0, 113.9, 109.9, 98.3, 83.0, 79.9, 73.4, 73.0, 72.9, 72.8, 72.1, 70.5, 69.4, 69.3, 62.9, 56.8, 55.8, 53.0, 49.7, 48.5, 45.6, 42.8, 41.6, 41.4, 37.8, 37.6, 35.0, 33.0, 32.0, 31.5, 30.9, 25.8, 15.2. HRMS-ESI (*m/z*): $[M]^{2+}$ calcd. for $C_{88}H_{107}N_{11}O_{18}S_3^{2+}$ 851.3490; found 851.3458.

Synthesis of compound 2c:

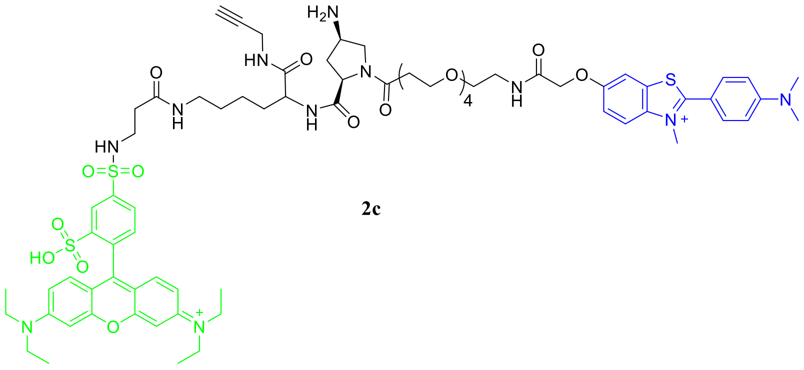

2c

Compound **22** (0.10 g, 0.06 mmol) was dissolved in 20% piperidine in DMF (3.5 mL). The reaction mixture was stirred for 1h at room temperature in the dark. After the reaction was completed (judging by neutral Al_2O_3 TLC plate, 8% methanol in DCM, R_f: 0.7 (compound **22**), 0.2 (product)), the solvent was removed in vacuum and residue was extracted with DCM (20.0 mL). The organic layer was dried over anhydrous sodium sulfate and evaporated to dryness. The crude product was purified by column chromatography (Neutral Al_2O_3, 7.5% MeOH in DCM) to afford **2c** as a pink solid (0.065 g, 75%). ^{1}H NMR (400 MHz, CD_3OD): δ 8.52 (s, 1H), 8.02 (d, *J* = 9.3 Hz, 1H), 8.01 (d, *J* = 9.3 Hz, 1H), 7.72 (br m, 3H), 7.49-7.42 (m, 2H), 7.04 (d, *J* = 9.5 Hz, 2H), 6.93 (d, *J* = 9.3 Hz, 4H), 6.82 (s, 2H), 4.61 (s, 2H), 4.51 (br d, 1H), 4.27-4.20 (m, 1H), 4.21 (s, 3H), 3.90-3.84 (m, 4H), 3.68 (t, *J* = 5.5 Hz, 2H), 3.60-3.48 (m, 24H), 3.4 (t, *J* = 5.5 Hz, 2H), 3.20 (q, *J* = 4.5 Hz, 3H), 3.09 (s, 6H), 2.81 (d, *J* = 9.0 Hz, 1H), 2.70-2.33 (m, 5H), 1.98 (d, *J* = 13.8 Hz, 1H), 1.85-1.63 (m, 2H), 1.48-1.37 (m, 4H), 1.21 (t, *J* = 6.7 Hz, 12H); ^{13}C NMR (100 MHz, CD_3OD): δ 174.6, 174.4, 174.3, 171.3, 160.5, 160.2, 158.9, 158.3, 156.9, 148.5, 144.7, 140.4, 136.7, 134.7, 134.9, 134.5, 133.9, 132.6, 130.3, 128.8, 121.6, 119.9, 116.5, 116.2, 114.4, 113.6, 109.7, 98.2, 81.6, 73.6, 72.8,72.7, 72.6, 72.5, 72.4, 71.6, 70.0, 61.8, 55.7, 53.4, 48.0, 42.0, 41.4, 41.2, 41.0, 40.1, 38.8, 37.4, 33.6, 30.9, 30.7, 25.1, 14.0. HRMS-ESI (m/z): $[M]^{2+}$ calcd. for $C_{73}H_{97}N_{11}O_{16}S_3^{2+}$ 739.8133; found 739.8107.

Synthesis of compound 2a:

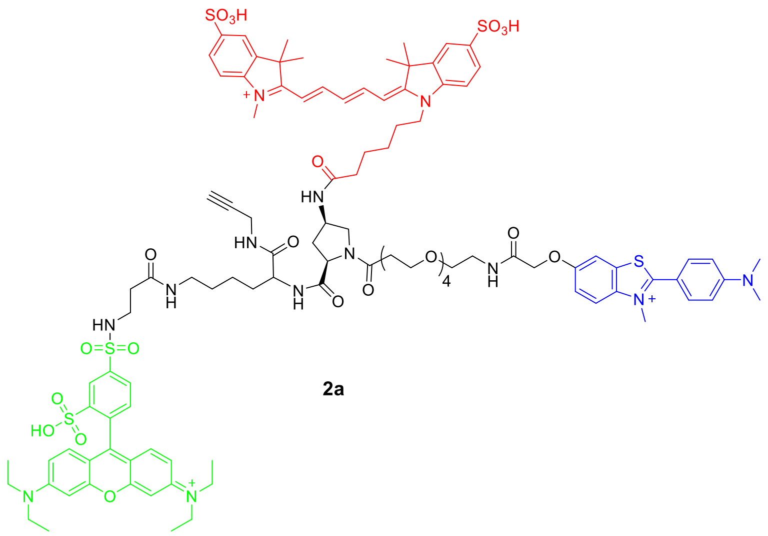

2a

Compound **2c** (30 mg, 0.02 mmol) was dissolved in DMSO (1.0 mL) and the solution was basified with DIPEA (4 µl, 23 µmol). Then compound **23** (18 mg, 24 µmol) in DMSO (0.50 mL) was added and the reaction mixture was stirred at room temperature. The reaction, monitored by HPLC, was completed in 2 h. The crude product material was purified by RP-HPLC (eluted at retention time 27 min) to afford a deep blue solid (14 mg, 30%). ^{1}H NMR (400 MHz, CD_3OD): *δ* 8.42 (s, 1H), 8.12-7.99 (m, 3H), 7.95-7.81 (m, 2H), 7.68 (s, 1H), 7.66 (s, 1H), 7.63 (t, *J* = 1.5 Hz, 1H), 7.60-7.55 (m, 3H), 7.34-7.25 (m, 2H), 7.10 (s, 1H), 7.08 (d, *J* = 1.5 Hz, 1H), 6.95 (s, 1H), 6.92 (s, 1H), 6.77 (d, *J* = 9.3 Hz, 4H), 6.67 (d, *J* = 1.9 Hz, 2H), 6.44 (t, *J* = 12.2 Hz, 1H), 6.13 (d, *J* = 9.8 Hz, 1H), 6.08 (d, *J* = 9.8 Hz, 1H), 4.46 (s, 2H), 4.29-4.23 (m, 1H), 4.14-4.09 (m, 1H), 4.06 (s, 3H), 3.88 (t, *J* = 6.7 Hz, 2H), 3.71 (q, *J* = 2.5 Hz, 2H), 3.55-3.48 (m, 2H), 3.46-3.30 (m, 24H), 3.28-3.16 (m, 4H), 3.29 (s, 3H), 3.06 (s, 2H), 2.95 (s, 6H), 2.93-2.90 (m, 1H), 2.46-2.30 (m, 3H), 2.26-2.10 (m, 3H), 1.94 (t, *J* = 7.0 Hz, 2H), 1.69-1.56 (m, 4H), 1.51 (s, 6H), 1.50 (s, 6H), 1.46-1.40 (m, 2H), 1.34-1.14 (m, 6H), 1.06 (t, *J* = 6.7 Hz, 12H); ^{13}C NMR (100 MHz, CD_3OD): δ 174.7, 174.4, 172.4, 170.0, 166.5, 160.4, 160.2, 158.1, 157.3, 156.6, 148.1, 145.7, 145.1, 144.3, 143.5, 140.4, 135.7, 135.2, 121.6, 120.9, 116.7, 116.5, 115.0, 114.0, 113.1, 110.7, 106.4, 98.3, 76.0, 72.8, 72.8, 72.7, 72.6, 71.8, 70.4, 69.3, 67.6, 55.6, 51.9, 51.8, 48.3, 43.5, 43.3, 43.0, 42.9, 30.2, 30.0, 29.7, 15.5. FT-IR: [cm^{-1}]: 3099, 2931, 2873, 1674, 1647, 1589, 1492, 1458, 1414, 1368, 1330, 1274, 1199, 1173, 1128, 1091, 1013, 925. HRMS-ESI (m/z): $[M]^{2+}$ calcd. for $C_{105}H_{133}N_{13}O_{23}S_5^{2+}$ 1051.9114; found 1051.9083.

Table S1: Preparative HPLC gradient for purification of **2a**

Time (minutes)	Eluent A (%)	Eluent B (%)
0.00	90	10
20	60	40
35	60	40
37	0	100
43	0	100
45	90	10
50	90	10

Synthesis of compound 2e (overview)

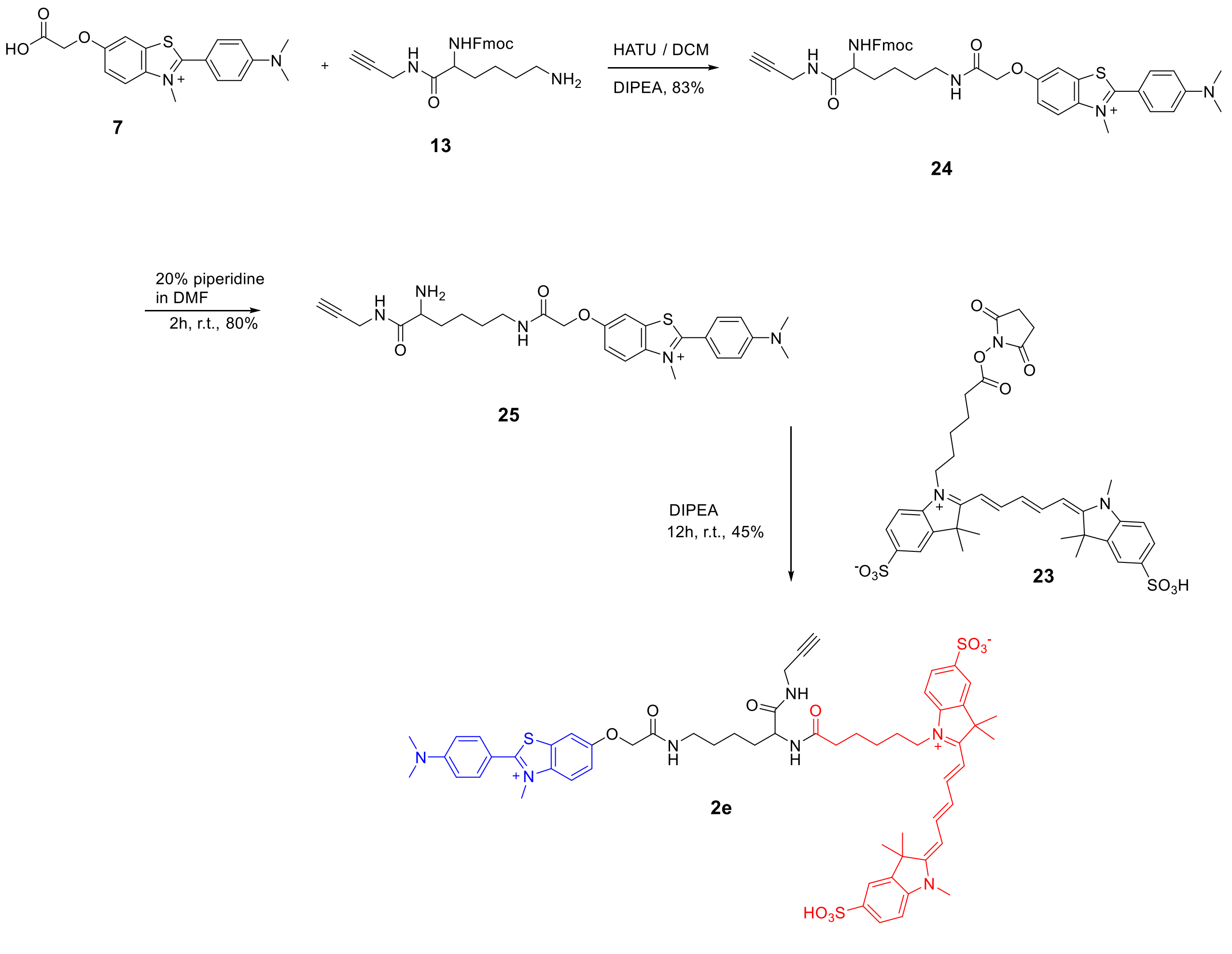

Synthesis of compound 24:

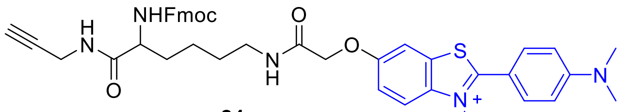

24

Compound **7** (0.10 g, 0.29 mmol) was dissolved in DCM (5.0 mL) and the solution was basified (judging by wet pH paper) with DIPEA (0.10 mL, 0.58 mmol). Coupling reagent HATU (0.22 g, 0.58 mmol) was added to the above reaction mixture which was stirred for 10 min at room temperature. Then compound **13** (0.12 g, 0.29 mmol) was added and the reaction was continued at room temperature for another 6h under argon. After the reaction was completed (judging by neutral Al_2O_3 TLC plate, 2% methanol in DCM, R_f: 0.1 (compound **13**), 0.6 (product)), the solvent was removed in vacuum and the residue was dissolved in DCM (10.0 mL) and washed with water (20.0 mL), brine (20.0 mL), and again with water (2 × 20.0 mL). The organic layer was dried over anhydrous sodium sulfate and evaporated to dryness. The crude product was purified by column chromatography (neutral Al_2O_3, 3% MeOH in DCM) to afford compound **24** (0.176 g, 83%). ^{1}H NMR (400 MHz, $(CD_3)_2SO$): δ 8.33 (t, J = 5.7 Hz, 1H), 8.21 (s, 1H), 8.15 (t, J = 9.2 Hz, 1H), 7.88-7.63 (m, 7H), 7.48 (d, J = 8.0 Hz, 2H), 7.40 (t, J = 7.4 Hz, 2H), 7.31 (t, J = 7.4 Hz, 2H), 6.95 (q, J = 9.2 Hz, 2H), 4.62 (t, J = 7.1 Hz, 2H), 4.30-4.09 (m, 6H), 3.90 (br d, 3H), 3.08 (br s, 9H), 1.60-1.10 (m, 6H); ^{13}C NMR (100 MHz, $(CD_3)_2SO$): δ 171.8, 171.6, 166.6, 157.2, 155.9, 154.5, 143.8, 143.7, 14.06, 137.3, 132.0, 129.5, 127.6, 127.0, 125.3, 120.0,118.5, 117.7, 111.9, 110.9, 107.5, 82.0, 73.0, 67.4, 66.6, 54.4, 53.6, 46.6, 40.2, 39.9, 39.8, 39.6, 38.2, 38.1, 31.5, 28.6, 27.9, 22.8. HRMS-ESI (*m/z*): $[M]^+$ calcd. for $C_{42}H_{44}N_5O_5S^+$ 730.3057; found 730.3047.

Synthesis of compound 25:

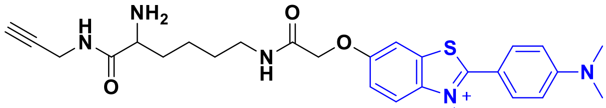

25

Compound **24** (0.10 g, 0.14 mmol) was dissolved in 20% piperidine in DMF (3.5 mL). The reaction mixture was stirred for 1h at room temperature in the dark. After the reaction was completed (judging by neutral Al_2O_3 TLC plate, 2% methanol in DCM, R_f: 0.7 (compound **24**), 0.4 (product)), the solvent was removed in vacuum and the residue was extracted with DCM (20.0 mL). The organic layer was dried over anhydrous sodium sulfate and evaporated to dryness. The crude product was purified by column chromatography (Neutral Al_2O_3, 7.5% MeOH in DCM) to afford compound **25** as a yellowish solid (0.065 g, 80%). ^{1}H NMR (400 MHz, MeOD): δ 8.0 (dd, J_1 = 9.3 Hz, J_2 = 3.2 Hz, 1H), 7.80-7.76 (m, 3H), 7.56 (dt, J_1 = 9.3 Hz, J_2 = 3.2 Hz, 1H), 6.98-6.94 (m, 2H), 4.66 (s, 2H), 4.28 (s, 3H), 3.99-3.97 (m, 2H), 3.35-3.30 (m, 3H), 3.16 (s, 6H), 2.62 (t, J = 2.6 Hz, 1H), 1.75-1.56 (m, 4H), 1.43-1.34 (m, 2H); ^{13}C NMR (100 MHz, MeOD): δ 177.4, 173.9, 169.8, 158.9, 155.5, 139.1, 133.1, 131.2, 120.2, 118.6, 113.2, 112.2, 108.2, 80.6, 72.3, 68.7, 55.8, 40.1, 39.9, 38.9, 38.8, 35.8, 30.1, 29.3, 23.8. HRMS-ESI (m/z): $[M]^+$ calcd. for $C_{27}H_{34}N_5O_3S^+$ 508.2376; found 508.2352.

Synthesis of compound 2e:

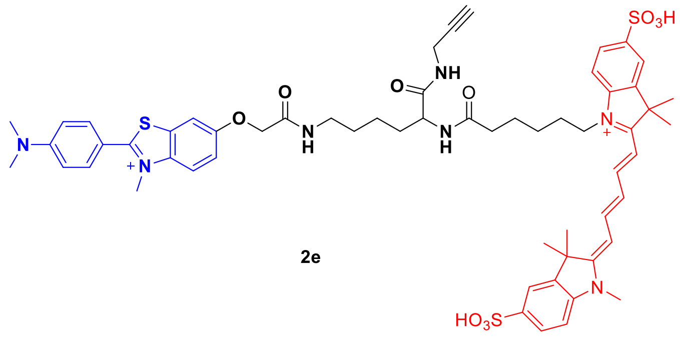

2e

Compound **25** (15 mg, 0.03 mmol) was dissolved in DMSO (1.0 mL) and the solution was basified with DIPEA (5 µl, 0.03 mmol). Then compound **23** (23 mg, 0.029 mmol) in DMSO (0.5 mL) was added and the reaction mixture was stirred at room temperature. The reaction, monitored by HPLC, was completed in 8 h. The crude material was purified by RP-HPLC (eluted at retention time 28 min) to afford a deep green solid (15 mg, 45%). ^{1}H NMR (400 MHz, $(CD_3)_2SO$): δ 8.39-8.27 (m, 3H), 8.20 (t, *J* = 5.9 Hz, 1H), 8.11 (d, *J* = 9.3 Hz, 1H), 7.87 (t, *J* = 2.6 Hz), 7.84 (s, 1H), 7.77 (s, 4H), 7.58 (t, *J* = 8.3 Hz), 7.43 (dd, J_1 = 9.3 Hz, J_2 = 2.6 Hz, 1H), 7.24-7.27 (m, 2H), 6.93 (d, *J* = 9.3 Hz, 3H), 6.52 (t, *J* = 12.4 Hz, 1H), 6.26 (d, *J* = 14 Hz, 1H), 6.21 (d, *J* = 14 Hz, 1H), 4.58 (s, 2H), 4.16 (s, 3H), 4.10-4.14 (m, 1H), 4.03 (t, *J* = 7.0 Hz, 2H), 3.80 (br s, 2H), 3.55 (s, 3H), 3.05 (s, 8H), 3.03 (s, 1H), 2.08 (t, *J* = 7.0 Hz, 1H), 1.64 (s, 12H), 1.55-1.11 (m, 12H); ^{13}C NMR (100 MHz, $(CD_3)_2SO$): δ 171.1, 174.3, 171.9, 171.6, 171.2, 166.3, 157.2, 156.7, 154.1, 153.5, 145.4, 145.1, 142.6, 141.9, 140.3, 140.1, 137.2, 132.0, 129.0, 125.5, 119.8, 118.4, 117.8, 111.9, 111.0, 110.0, 107.0, 81.1, 72.9, 69.9, 67.3, 52.0, 50.29, 48.8, 40.1, 39.9, 39.7, 39.5, 38.1, 28.6, 27.8, 27.0, 26.9, 25.5, 24.7. HRMS-ESI (m/z): $[M]^{2+}$ calcd. for $C_{59}H_{71}N_7O_{10}S_3^{2+}$ 566.7206; found 566.7189.

Table S2: Preparative HPLC gradient for purification of **2e**

Time (minutes)	Eluent A (%)	Eluent B (%)
0.00	90	10
10	75	25
25	60	40
32	60	40
34	0	100
38	0	100
39	90	10
45	90	10

Figures and Tables

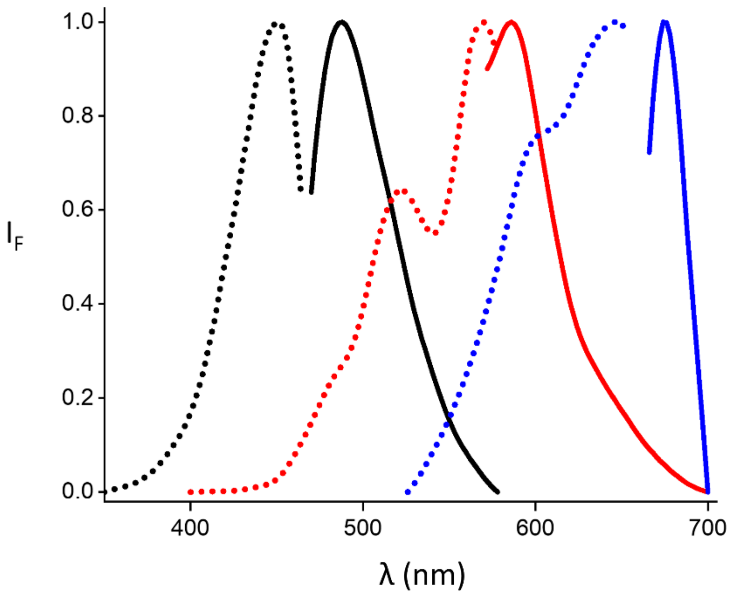

Figure S1. Normalized excitation (dotted line) and emission spectra (solid line) of ThT (with replicator fibers (**1a**)$_6$, black line), SRB (red line), and sCy5 (blue line).

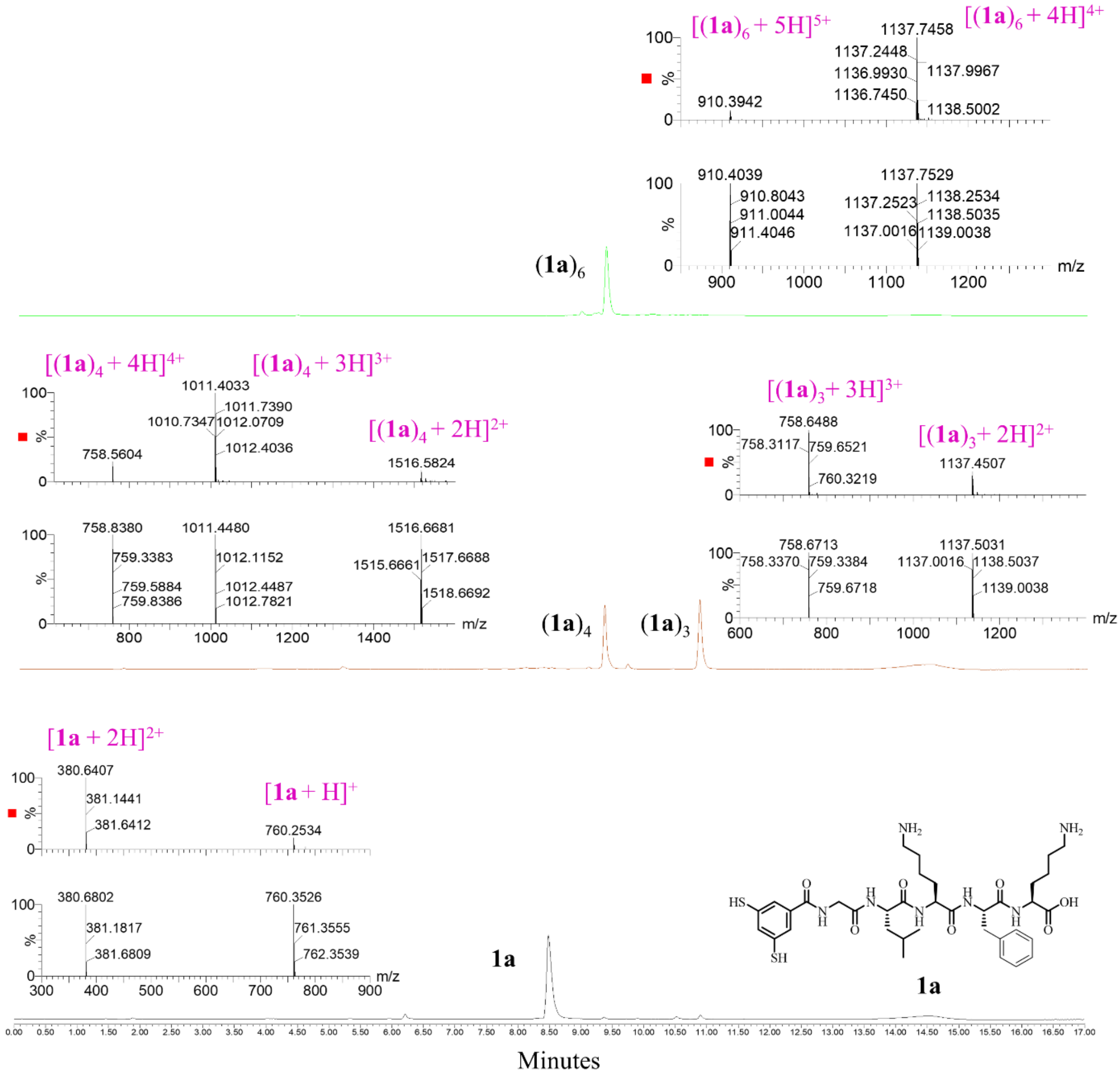

Figure S2. UPLC traces (monitored at 254 nm) and mass spectra (bottom and top panels show theoretical and observed mass, respectively) of samples dominated by monomers **1a**, mixture of trimers-tetramers (**1a**)$_3$/(**1a**)$_4$), and fibers (**1a**)$_6$ prepared from building **1a** using the protocol described in the methods section (main text).

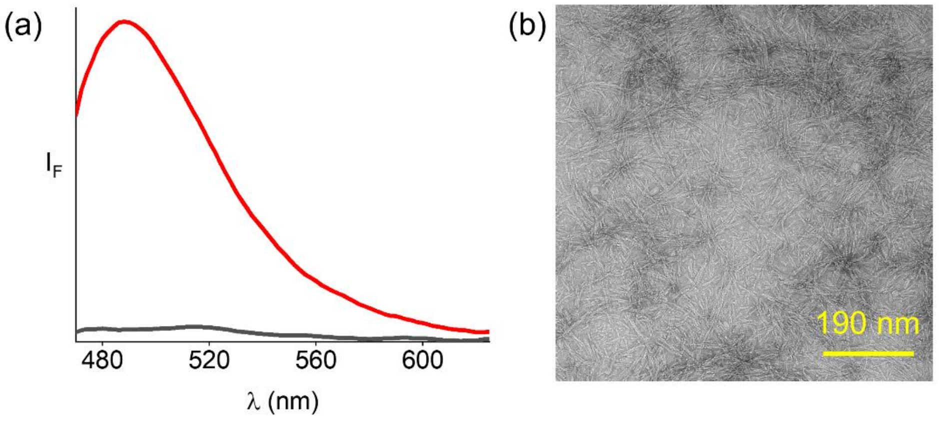

Figure S3. (a) Emission spectra of ThT (2.0 µM, black line, 50 mM borate buffer) in the presence of fibers (**1a**)$_6$ (30 µM in building block **1a**, red line); (b) Corresponding negative straining TEM image.

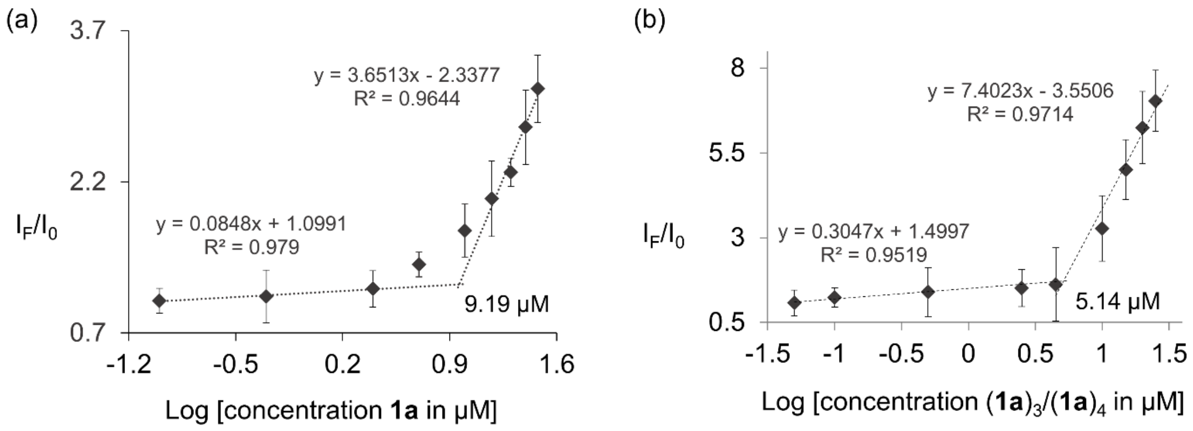

Figure S4. Determination of critical aggregation concentration (CAC) by titration **2a** (2.0 µM, 50 mM borate buffer, pH 8.2) with different concentration of (a) monomers **1a**, and (b) mixture of trimers-tetramers (**1a**)$_3$/(**1a**)$_4$ (50 mM borate buffer, pH 8.2). Concentrations are given in units of building block **1a**.

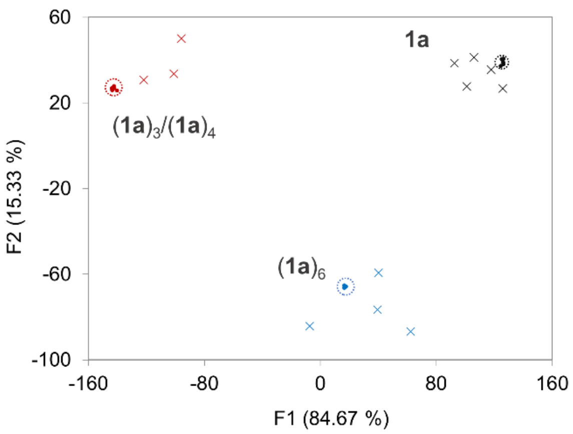

Figure S5. PCA plot (training set) which allowed the collect identification of 12 out of 12 unknown samples of monomers **1a**, mixture of trimers-tetramers (**1a**)$_3$/(**1a**)$_4$ and fibers (**1a**)$_6$ (**Table S3**) prepared from building block **1a**. Training set and unknown samples are denoted by circles and crosses respectively.

Table S3. LDA prediction of the identity of unknown samples.

Sample	Identity	F1	F2	Predicted
1	(**1a**)$_3$/(**1a**)$_4$	-101.096	33.605	(**1a**)$_3$/(**1a**)$_4$
2	(**1a**)$_3$/(**1a**)$_4$	-95.889	50.153	(**1a**)$_3$/(**1a**)$_4$
3	**1a**	101.063	27.734	**1a**
4	**1a**	117.912	35.618	**1a**
5	**1a**	125.793	26.775	**1a**
6	(**1a**)$_3$/(**1a**)$_4$	-121.813	30.725	(**1a**)$_3$/(**1a**)$_4$
7	(**1a**)$_6$	62.329	-86.815	(**1a**)$_6$
8	**1a**	105.914	41.332	**1a**
9	(**1a**)$_6$	40.116	-59.238	(**1a**)$_6$
10	**1a**	92.530	38.631	**1a**
11	(**1a**)$_6$	39.385	-76.594	(**1a**)$_6$
12	(**1a**)$_6$	-7.267	-84.187	(**1a**)$_6$

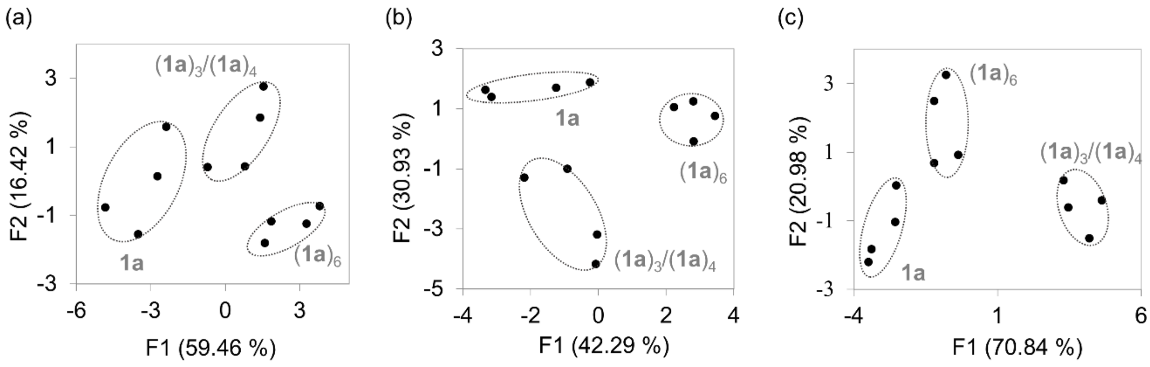

Figure S6. PCA generated using the changes of emission of **2a** (2.0 µM, 50 mM borate buffer, pH 8.2) in presence of (a) 300 nM, (b) 3.0 µM, (c) 10 µM of monomers **1a**, mixture of trimers-tetramers (**1a**)$_3$/(**1a**)$_4$, and fibers (**1a**)$_6$ as described in the experimental section. Concentrations are given in units of building block **1a**

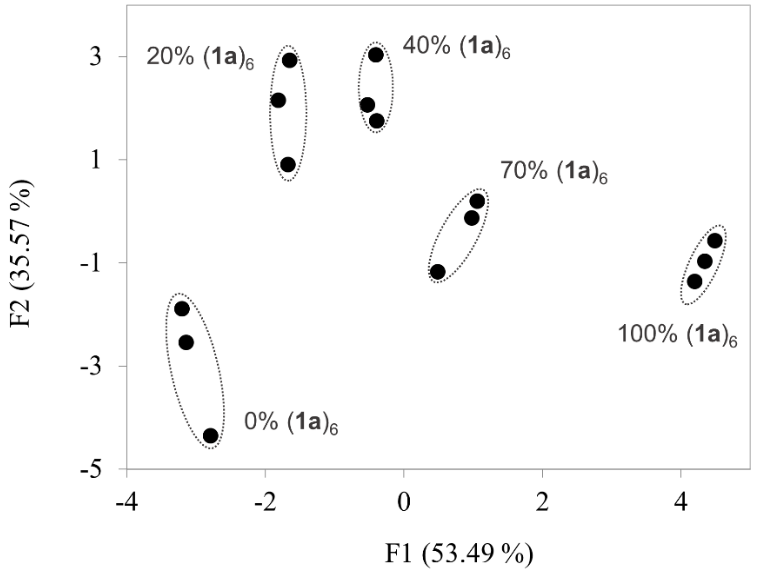

Figure S7. PCA of emission patterns (λ_{ex} = 440 nm and λ_{ex} = 530 nm) obtained from the mixture of **2a** (2.0 µM) and the samples shown in **Table 1** (main text).

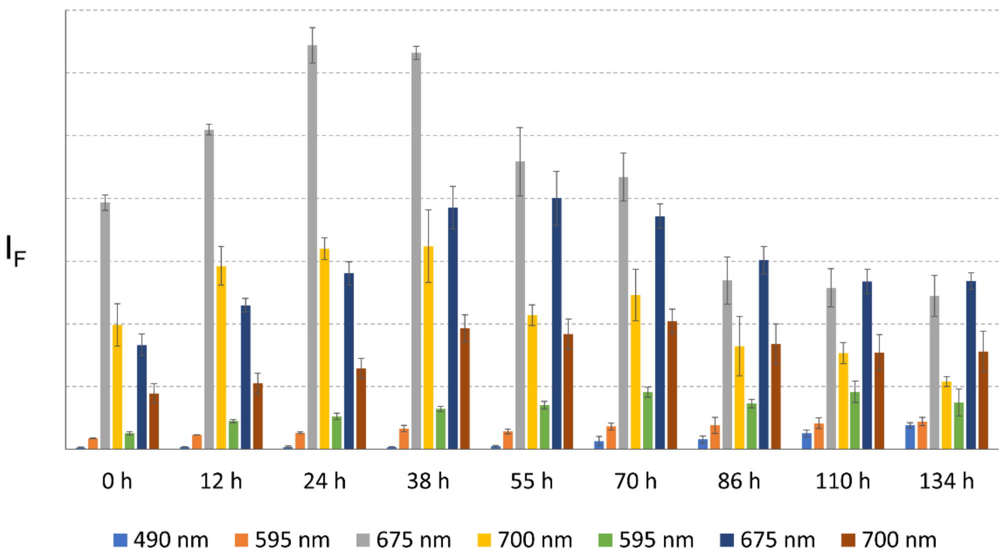

Figure S8. Change in fluorescence intensity of **2a** at seven different emission channels (λ_{ex} = 440 nm and 530 nm, respectively) in a mixture made from building block **1a** (30 µM in building block **1a**) co-incubated with sensor **2a** (2.0 µM) in borate buffer (50 mM in boron atoms, pH 8.2, stirred at 1200 rpm at 30 °C

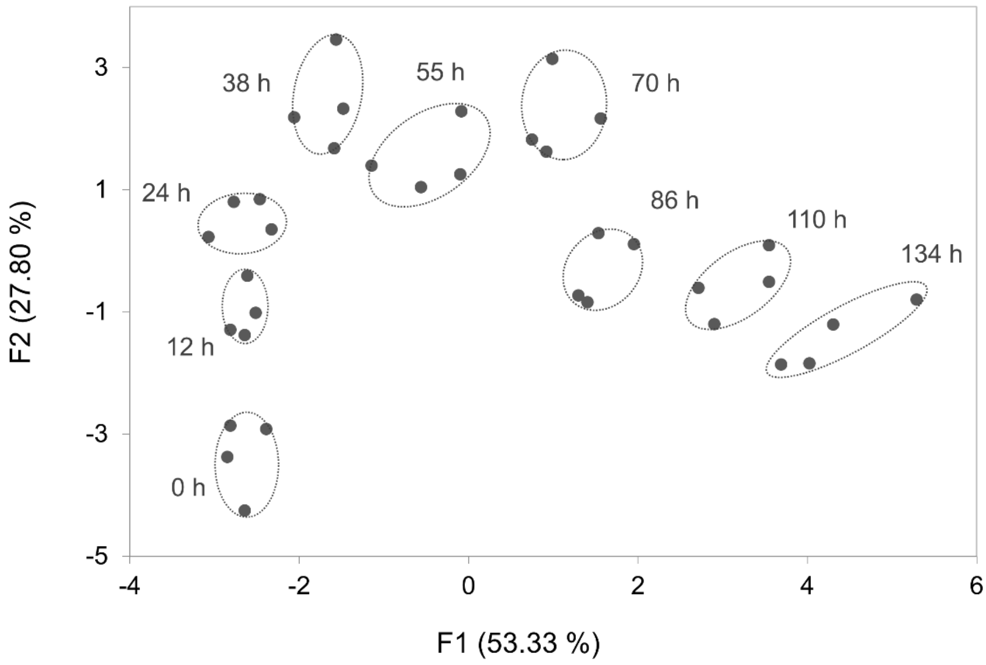

Figure S9. PCA of the emission data of **2a** (2.0 µM) at various time point (0 to 134 h) in a mixture prepared from **2a** (2.0 µM) and **1a** (30 µM in units of building block) co-incubated in borate buffer (50 mM, pH 8.2) and stirred 1200 rpm at 30 °C.

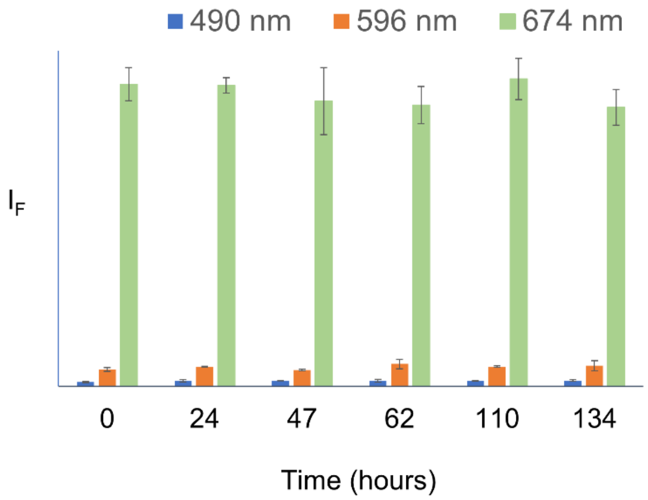

Figure S10. Emission of **2a** (2.0 μM) stirred at 1200 rpm in borate buffer (50 mM, pH 8.2) at 30 °C recorded at various time points. The emission of **2a** did not change over time supporting the suitability of **2a** for the real-time tracking of self-replicator.

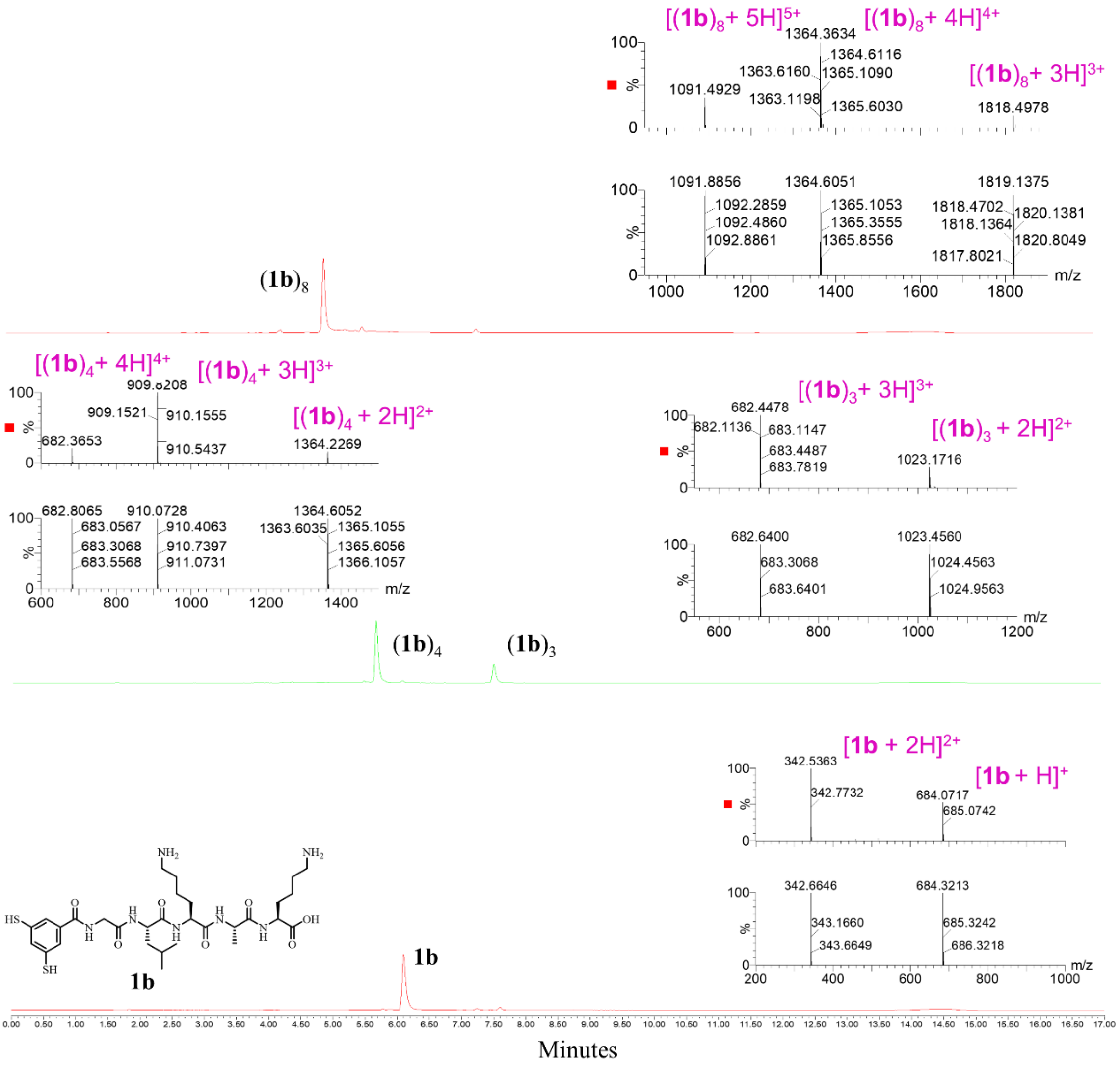

Figure S11. UPLC traces (monitored at 254 nm) and mass spectra (bottom and top panels show theoretical and observed mass, respectively) recorded for monomers **1b**, mixture of trimers-tetramers (**1b**)$_3$/(**1b**)$_4$, and fibers (**1b**)$_8$ prepared from building **1b** using the protocol described in the methods section (main text).

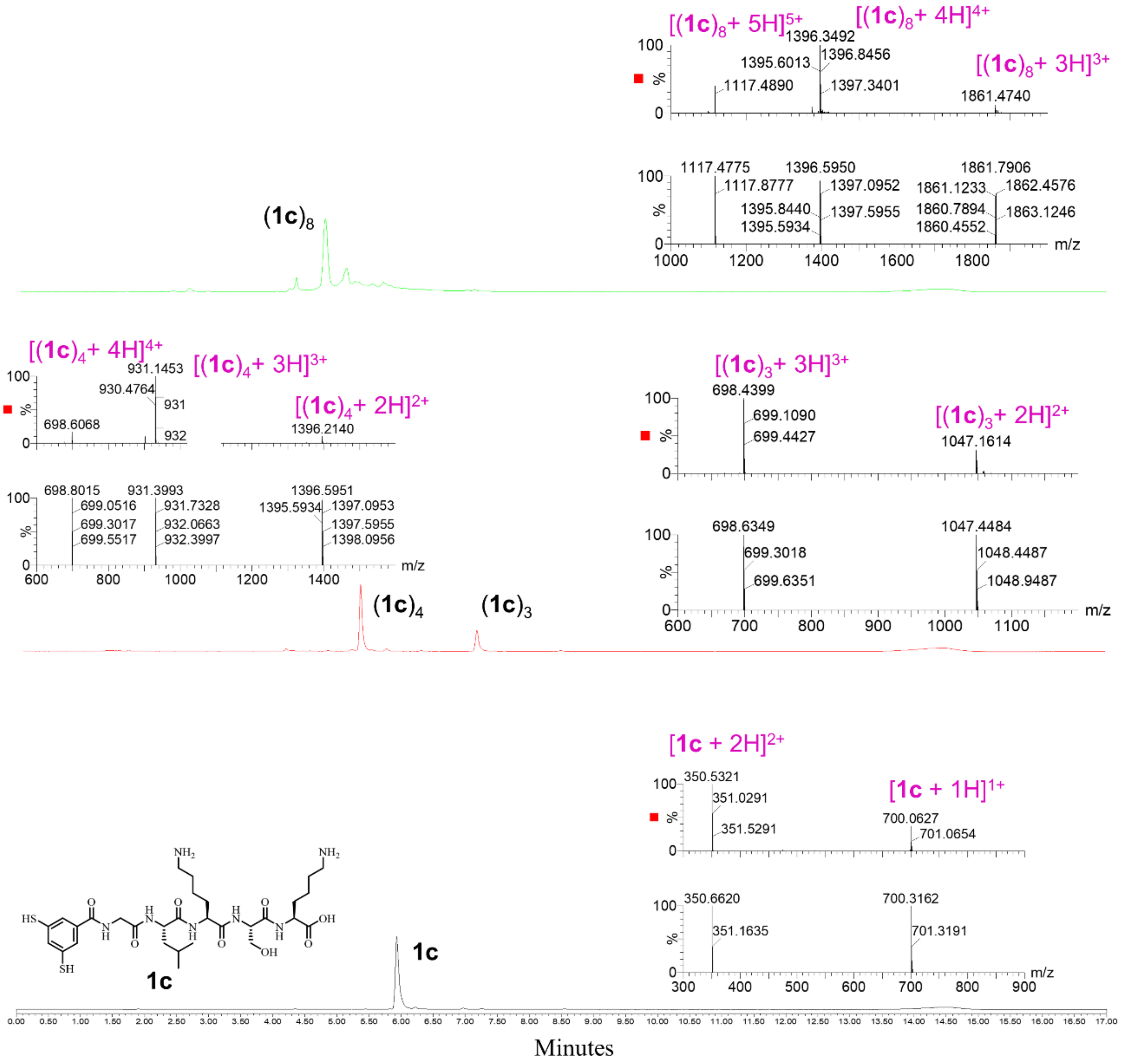

Figure S12. UPLC traces (monitored at 254 nm) and mass spectra (bottom and top panels show theoretical and observed mass, respectively) recorded for monomers **1c**, mixture of trimers-tetramers (**1c**)$_3$/(**1c**)$_4$, and fibers (**1c**)$_8$ prepared from building **1c** using the protocol described in the methods section (main text).

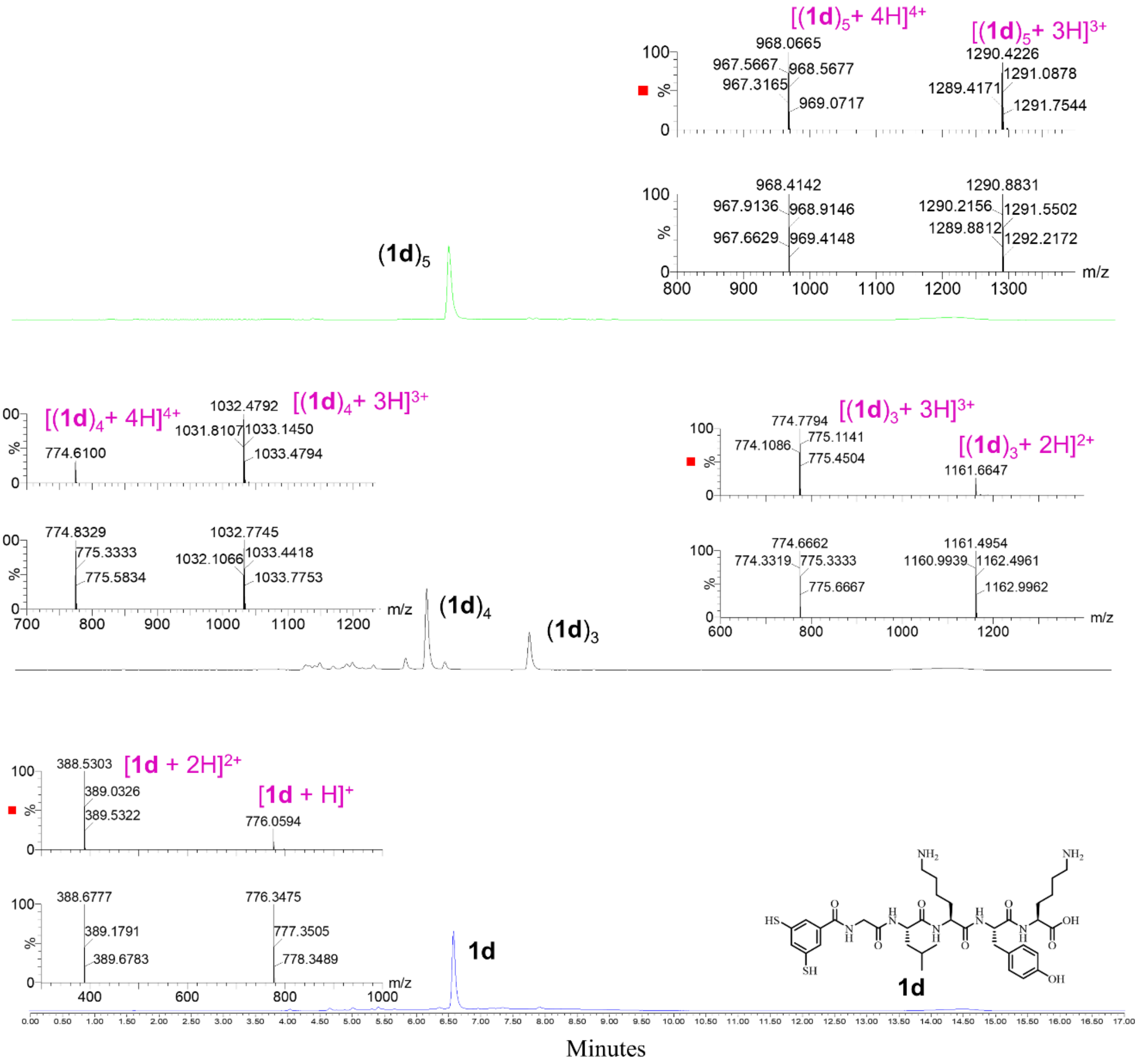

Figure S13. UPLC traces (monitored at 254 nm) and mass spectra (bottom and top panels show theoretical and observed mass, respectively) recorded for monomers **1d**, mixture of trimers-tetramers (**1d**)$_3$/(**1d**)$_4$, and fibers (**1d**)$_5$ prepared from building **1d** using the protocol described in the methods section (main text).

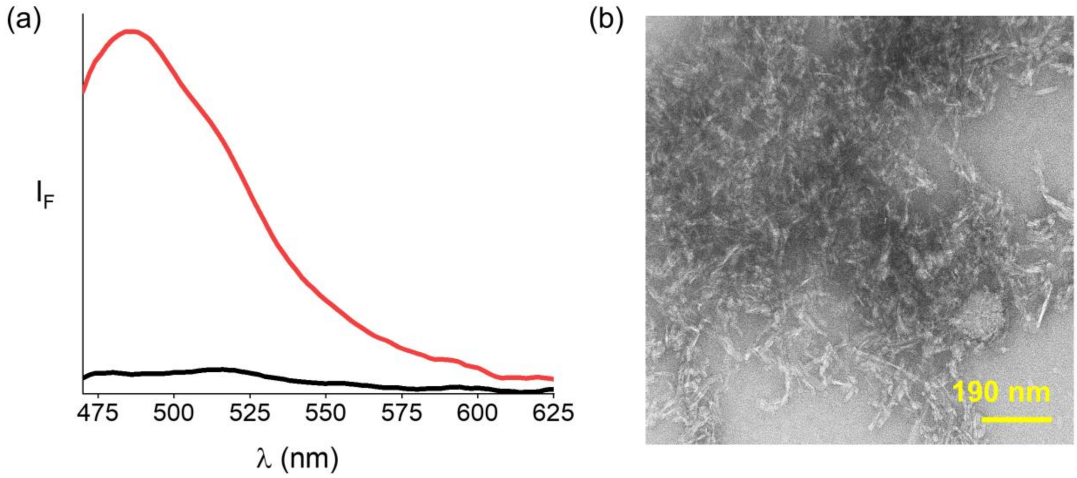

Figure S14. (a) Emission spectra of ThT (2.0 µM, black line, 50 mM borate buffer) in the presence of fibers (30 µM in units of building block, red line) prepared from peptide building blocks **1b** (2.0 mM in units of building block); (b) Corresponding TEM image.

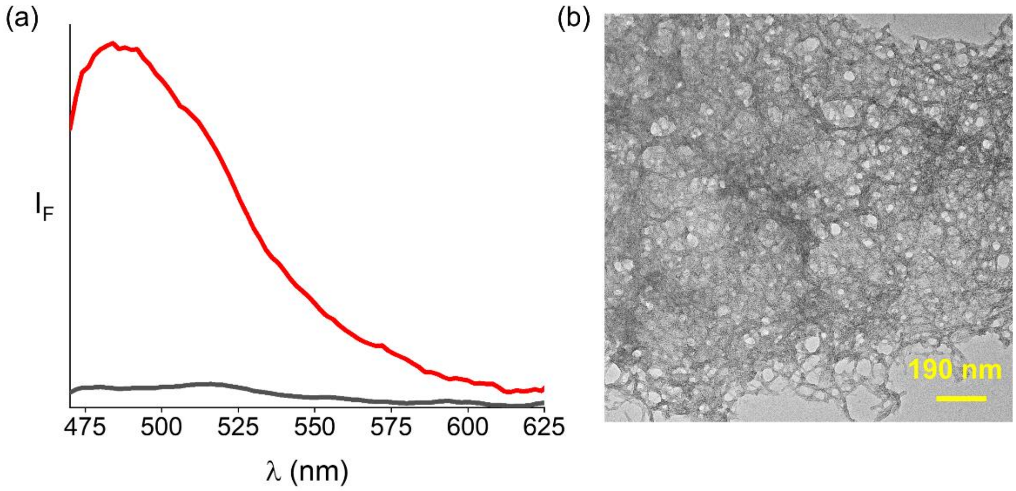

Figure S15. (a) Emission spectra of ThT (2.0 µM, black line, 50 mM borate buffer) in the presence of fibers (30 µM in units of building block, red line) prepared from peptide building blocks **1c** (2.0 mM in units of building block); (b) Corresponding TEM image.

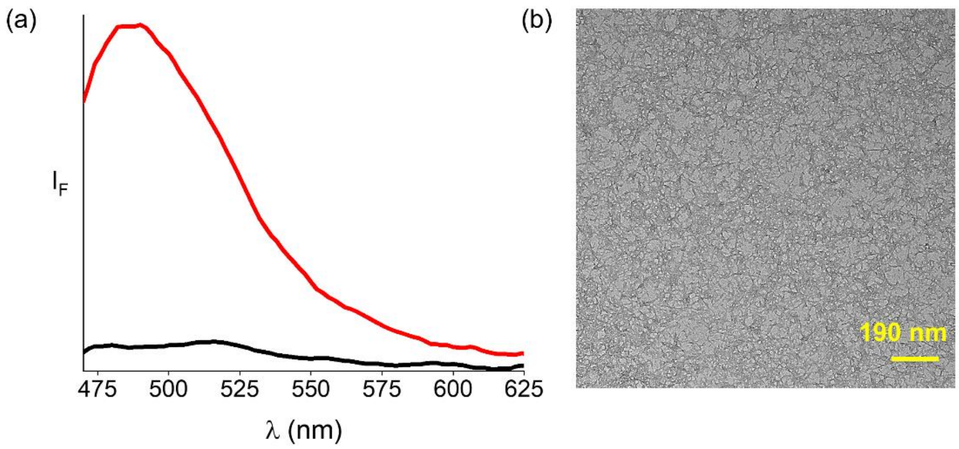

Figure S16. (a) Emission spectra of ThT (2.0 µM, black line, 50 mM borate buffer) in the presence of fibers (30 µM in units of building block, red line) prepared from peptide building blocks **1d** (2.0 mM in units of building block); (b) Corresponding TEM image.

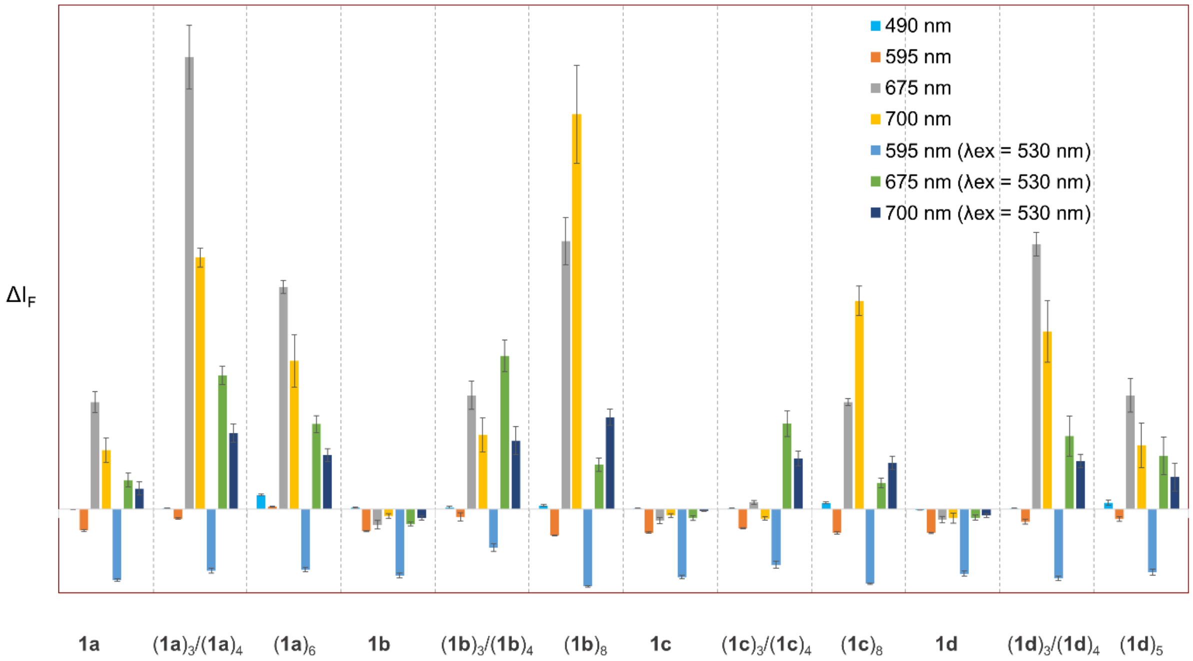

Figure S17. Emission data obtained from **2a** (2.0 µM) at seven different emission channels (λ_{ex} = 440 nm and λ_{ex} = 530 nm) in response to **1a**, **(1a)**$_3$/**(1a)**$_4$, **(1a)**$_6$, **1b**, **(1b)**$_3$/**(1b)**$_4$, **(1b)**$_8$, **1c**, **(1c)**$_3$/**(1c)**$_4$, **(1c)**$_8$, **1d**, **(1d)**$_3$/**(1d)**$_4$, **(1d)**$_5$ prepared from building block **1a-d** in 50 mM borate buffer, pH 8.2.

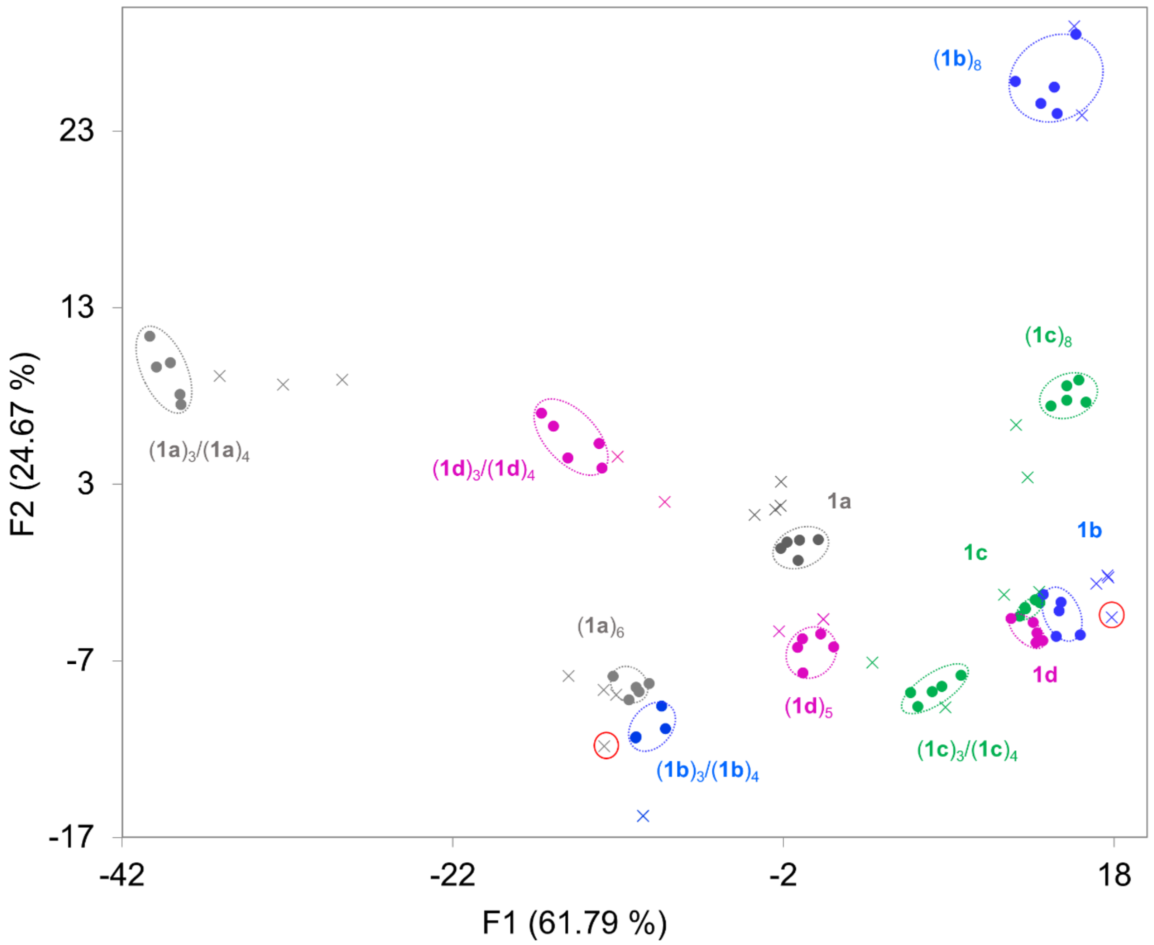

Figure S18. LDA plot (training set) used to identify 26 out of 28 unknown samples of monomers, mixture of trimers-tetramers, and fibers (**Table S4**) prepared from building blocks **1a-d**. Training set and unknown samples are denoted by circles and triangle respectively. Misclassified data points are shown in red circle.

Table S4. LDA prediction of the identity of unknown samples.

Sample	Identity	F1	F2	Predicted
1	$(\mathbf{1c})_3$/$(\mathbf{1c})_4$	3.369	-7.104	$(\mathbf{1c})_3$/$(\mathbf{1c})_4$
2	$(\mathbf{1c})_3$/$(\mathbf{1c})_4$	7.801	-9.628	$(\mathbf{1c})_3$/$(\mathbf{1c})_4$
3	**1a**	-3.732	1.276	**1a**
4	**1b**	17.608	-2.158	**1b**
5	**1a**	-2.189	1.769	**1a**
6	$(\mathbf{1d})_3$/$(\mathbf{1d})_4$	-12.031	4.565	$(\mathbf{1d})_3$/$(\mathbf{1d})_4$
7	$(\mathbf{1a})_6$	-12.888	-8.652	$(\mathbf{1a})_6$
8	**1c**	13.479	-3.090	**1c**
9	$(\mathbf{1a})_3$/$(\mathbf{1a})_4$	-36.104	9.134	$(\mathbf{1a})_3$/$(\mathbf{1a})_4$
10	$(\mathbf{1c})_8$	12.071	6.355	$(\mathbf{1c})_8$
11	$(\mathbf{1d})_5$	0.417	-4.651	$(\mathbf{1d})_5$
12	$(\mathbf{1b})_8$	16.053	23.888	$(\mathbf{1b})_8$
13	$(\mathbf{1d})_3$/$(\mathbf{1d})_4$	-9.191	1.997	$(\mathbf{1d})_3$/$(\mathbf{1d})_4$
14	$(\mathbf{1a})_3$/$(\mathbf{1a})_4$	-32.258	8.629	$(\mathbf{1a})_3$/$(\mathbf{1a})_4$
15	**1d**	17.856	-4.526	**1b**
16	**1a**	-2.141	3.144	**1a**
17	**1a**	-2.495	1.568	**1a**
18	$(\mathbf{1b})_8$	15.598	28.928	$(\mathbf{1b})_8$
19	$(\mathbf{1b})_3$/$(\mathbf{1b})_4$	-12.856	-11.834	$(\mathbf{1a})_6$
20	**1c**	11.332	-3.255	**1c**
21	$(\mathbf{1d})_5$	-2.272	-5.328	$(\mathbf{1d})_5$
22	**1b**	17.649	-2.264	**1b**
23	$(\mathbf{1b})_3$/$(\mathbf{1b})_4$	-10.501	-15.781	$(\mathbf{1b})_3$/$(\mathbf{1b})_4$
24	$(\mathbf{1c})_8$	12.766	3.398	$(\mathbf{1c})_8$
25	$(\mathbf{1a})_3$/$(\mathbf{1a})_4$	-28.685	8.904	$(\mathbf{1a})_3$/$(\mathbf{1a})_4$
26	**1b**	16.933	-2.641	**1b**
27	$(\mathbf{1a})_6$	-12.123	-8.914	$(\mathbf{1a})_6$
28	$(\mathbf{1a})_6$	-14.999	-7.864	$(\mathbf{1a})_6$

References

[1] Otto, S.; Furlan, R.; Sanders, J. Selection and amplification of hosts from dynamic combinatorial libraries of macrocyclic disulfides. Science **2002**, *297*, 590–593.
[2] Qin, L.; Vastl, J.; Gao, J. Highly sensitive amyloid detection enabled by thioflavin T dimers. *Mol. BioSyst.* **2010**, 6, 1791-1795.
[3] Jose, J.; Loudet, A.; Ueno, Y.; Barhoumi, R.; Burghardt, R. C.; Burgess, K. Intracellular imaging of organelles with new water-soluble benzophenoxazinedyes. *Org. Biomol. Chem.* **2010**, *8*, 2052-2059.
